# Clovis points and foreshafts under braced weapon compression: Modeling Pleistocene megafauna encounters with a lithic pike

**DOI:** 10.1371/journal.pone.0307996

**Published:** 2024-08-21

**Authors:** R. Scott Byram, Kent G. Lightfoot, Jun Ueno Sunseri

**Affiliations:** 1 Archaeological Research Facility, University of California, Berkeley, Berkeley, California, United States of America; 2 Department of Anthropology, University of California, Berkeley, Berkeley, California, United States of America; Tel Aviv university, ISRAEL

## Abstract

Historical and ethnographic sources depict use of portable braced shaft weapons, or pikes, in megafauna hunting and defense during Late Holocene millennia in North and South America, Africa, Eurasia and Southeast Asia. Given the predominance of megafauna in Late Pleistocene North America during the centuries when Clovis points appeared and spread across much of the continent (13,050–12,650 cal BP), braced weapons may have been used in hunting of megaherbivores and defense against megacarnivores. Drawing from historical examples of pike use against lions, jaguars, boars, grizzlies, carabao and warhorses we consider the possibility of a fluted lithic pike. Associated osseous rods have been problematic as Clovis foreshafts due to the bevel angle and the apparent weakness of the splint haft when great strength is needed for deep penetration in megafauna hunting. However our review of Late Holocene pike use in megafauna encounters indicates the sharp tip becomes less important after hide or armor has been pierced because compression is sustained. Thus, foreshaft collapse after hide entry may not limit but rather increase the efficacy of the braced weapon. We conduct preliminary static experiments to model a fluted pike that adjusts during compression such that haft collapse and point detachment (when point jams on impact with bone) preserve the fluted biface, beveled rod and wooden mainshaft tip. In addition to Clovis point attributes and association with osseous rods, potential archaeological correlates of Clovis pike use include the high frequency of Clovis point isolates and concentrations of complete points with unbutchered mammoth remains at sites such as Naco in Arizona.

## 1. Introduction

### 1.1 Overview

**Fig 1:**
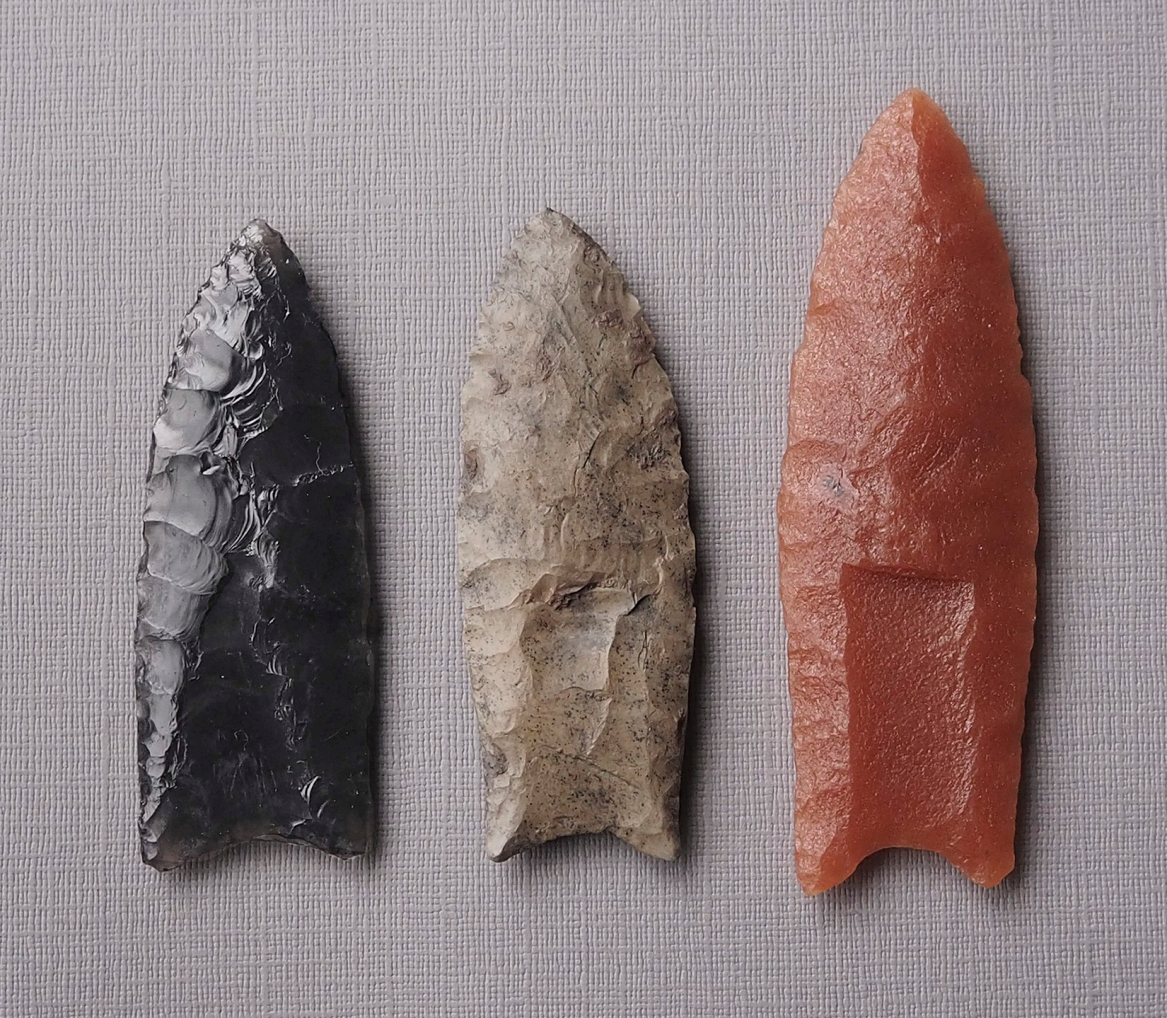
Clovis points with distinctive flute or channel flake scar in basal area. S. Byram photo of casts.

Fluted stone bifaces known as Clovis points appeared and spread across vast regions of North American megafauna habitat during four centuries of the terminal Pleistocene [1]. Several decades of academic research have illuminated many aspects of Clovis technology, but there is no consensus on the technological basis for the distinctive Clovis point (Fig 1), its robust, finely worked tip and midsection and its comparatively gracile, fluted base [2–4]. This paper considers the practicality of large mammal confrontation with sharp-tipped shaft weapons to assess whether fluted Clovis points and osseous rods may have tipped pikes used for hunting megafauna much larger than humans.

**Fig 2:**
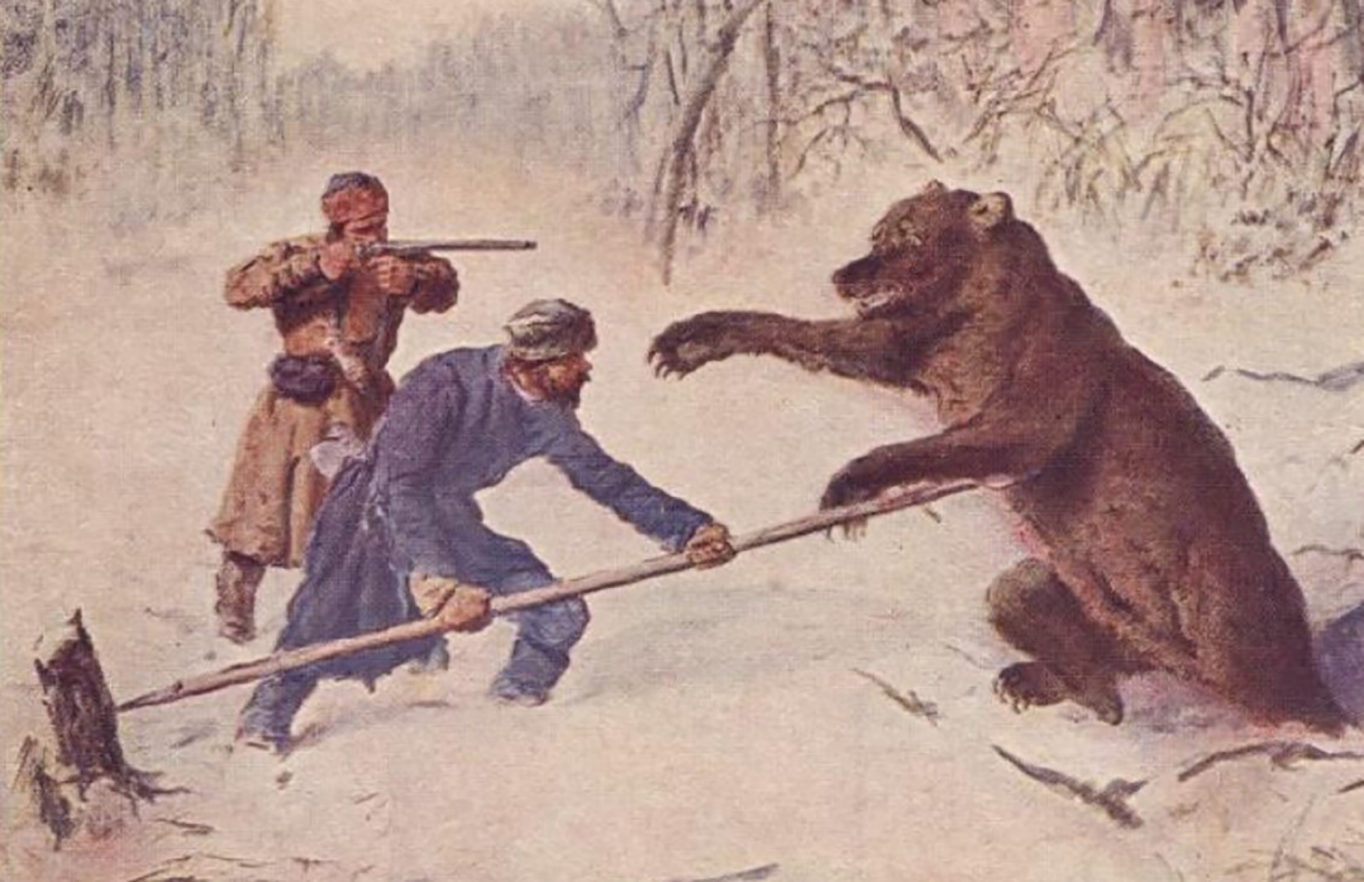
Braced weapon bear hunting in Northern Eurasia, 19^th^ century. Pavlov Sokolov. Blavatnik Foundation Leningrad Collection http://n2t.net/ark:/86084/b49b7w.

Ethnographic studies of weapons used in megafauna hunting have been invaluable in assessing Pleistocene hunting techniques. However as Annemieke Milks [5:1] notes, “selective references in relation to the use of wooden spears have overlooked additional examples that point to a richness and variability of technology and behaviour that is invisible in the Pleistocene archaeological record.” Fluted point studies and paleolithic studies in general often consider bifacial points as tipping the thrusting spear, hand thrown spear, and complex projectiles such as the spear launched with an atlatl, techniques well-documented historically and through experimentation [2, 6–8]. However there are also abundant historical depictions of non-projectile, braced weapon use against mammals a quarter ton or larger that charge or lunge at humans when being hunted (Fig 2), and large predators attacking people or their livestock. These accounts demonstrate that for millennia, including centuries of firearm use, megafauna such as brown bears, lions, jaguars, boars, carabao and warhorses were often slain with a braced piercing weapon, sometimes known as a pike, that uses the animal’s momentum to impale the animal and arrest its movement toward the pike wielder. Pike impalement often causes more massive injury than a thrust or launched spear can produce.

Despite the effectiveness of pikes historically, braced weapon lithic technology has seen very limited research to date. Specific instances of the technique appear in 20^th^ century ethnographies [9, 10]. Washburn [11:153] considered impalement a hunting technique that made it possible for humans to hunt even the largest terrestrial animals while maintaining their safety, providing an example in of Alutiiq hunters slaying bears over ten times their size with braced spear impalement. Adamson [12:146] described braced weapon use against lions by the Maasai and suggested that braced spear use may have begun during the Pleistocene. Fedje [13:25] and McLaren et al. [14:9] addressed possible Early Holocene archaeological braced lithic weapons based on their association with bear caves on the Northwest Coast. Our 2023 SAA paper on Clovis pikes [15] introduced the possibility of Clovis braced weapons in hunting and defense, and Baldino et al. [16:2] discussed the need for experimentation with defensive braced spear use. However a possible relationship between fluted points and osseous rods in braced weapon use has not previously been addressed through experimentation.

In order to assess variability in pike hunting and defense, we begin by examining records of historical braced weapon use against large mammals. We were unable to identify previous comparative research on pike hunting, so our findings on this topic draw from a wide variety of sources including ethnographies, historical accounts of hunting practices, and historical artwork depicting megafauna encounters with pikes. Pike use against warhorses and troops in military encounters is more extensively documented [17–20], and we have examined these sources as well as the period observations of Smythe [21], Ptolemy I [22:71], and Xenophon [23].

**Fig 3:**
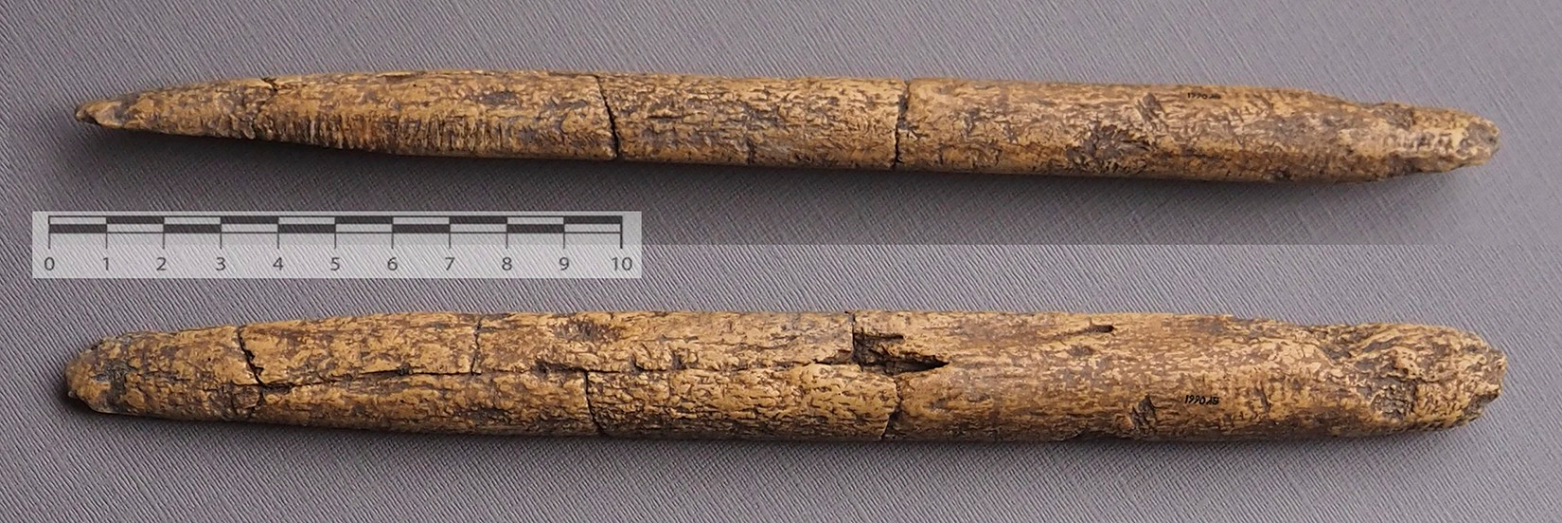
Two views of a reassembled beveled bone rod or foreshaft from the East Wenatchee site [24]; S. Byram photos of cast #P-26, Lithic Casting Labs, Troy, Illinois.

Based on patterns in the historical data on pike use in megafauna encounters, we consider the possibility that the distinctive characteristics of Clovis points relate to their use as lithic pike points. We examine characteristics of archaeological Clovis points, associated beveled osseous rods (Fig 3), which could also have been made of wood, and likely characteristics of pike shafts to assess their suitability for a braced weapon strategy. We present findings from three static experiments involving Clovis points and beveled rods in a proposed pike tip configuration and discuss the archaeological implications of these results.

### 1.2 Historical accounts of pike hunting

**Fig 4:**
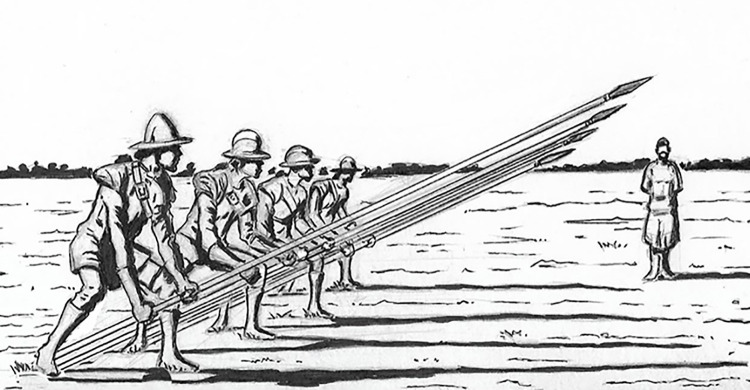
Pikes set in “charge for horse” position (based on Smythe [21:f7]). Original drawing, Ronald R. Nelson.

First identified with mammoth remains in the 1930s [25], Clovis points are often called *lanceolate* based on their lance point shape. Lances are well known as polearms used by cavalry in many regions. The foot soldier’s version of the lance is called a pike [20]. The key elements of the pike are a sharp tip for entering thick hide or armor and a long, sturdy shaft that could be braced in the ground to receive a charge with deadly force resistance (Fig 4). Pikes were used for well over two millennia to stop charging warhorses in battle, and they can also be used as a long thrusting spear [17–19].

**Fig 5:**
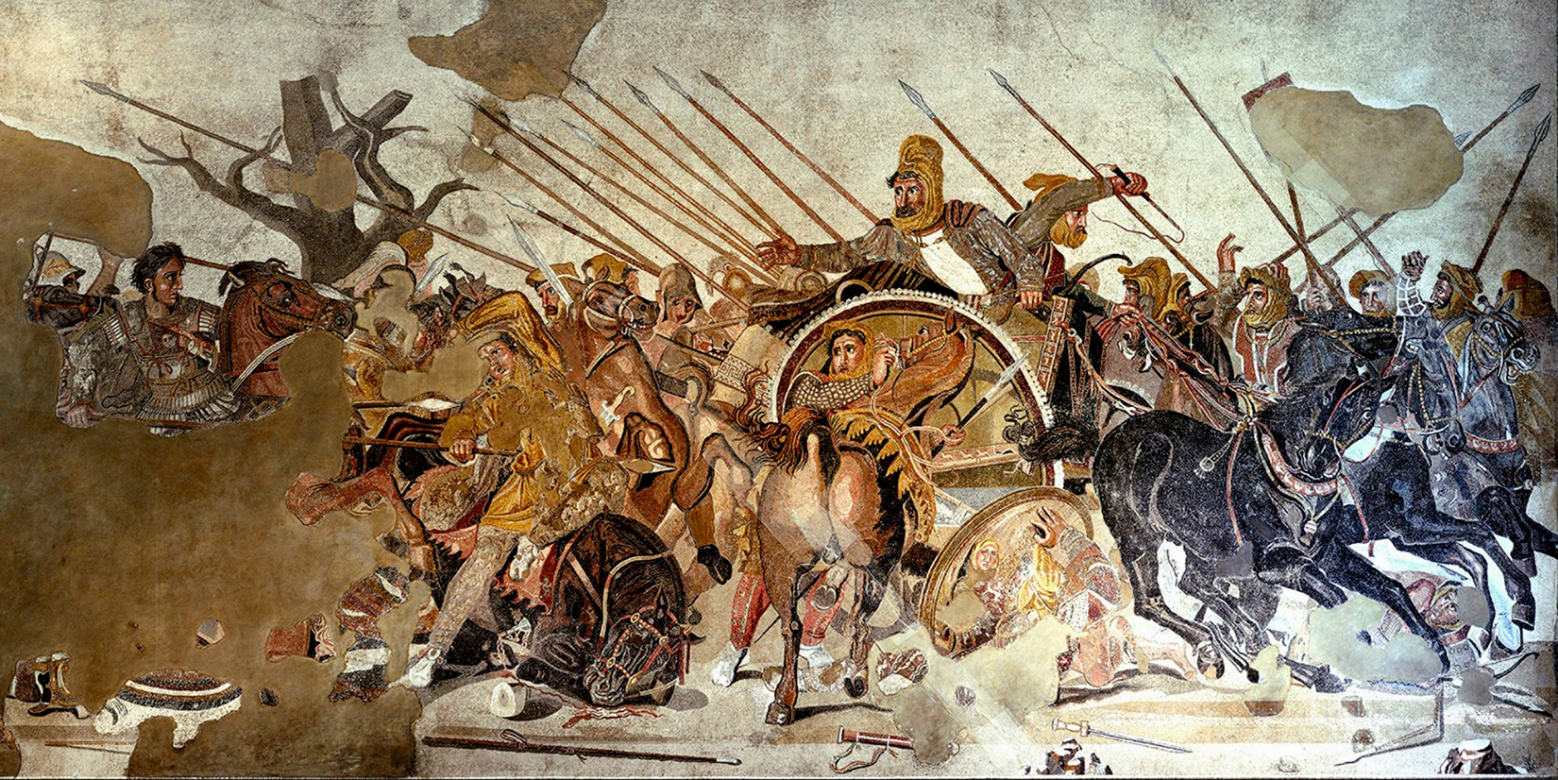
Greek sarissa pikes used in the Battle of Issus, 333 B.C.E.; Alexander Mosaic, House of the Faun, Pompeii. Berthold Werner, Public domain, via Wikimedia Commons.

While accounts of pikes used against charging horses are numerous, there are also many depictions of piercing weapons used in braced position for hunting animals such as boar [17, 18]. Eurasian wild boars can grow to over 300 kg, and they often charge when confronted. Hunters risked being severely wounded by tusks in these encounters, so the boar hunting pike was designed to stop the impaled animal from moving down the shaft to reach the hunter before it expires. According to Greek historian Xenophon, the well-known Greek sarissa pike (Fig 5) may have developed from the ancient boar hunting spear [18:397, 23:10.16], usually with a smaller metal pike head of bronze or iron [19]. The Macedonians often used braced sarissa pikes against cavalry. Ptolemy and others used pikes against Indian and African war elephants [22:70, 26:226], though we have not identified detailed accounts of a braced pike technique used in proboscidean encounters. Agam and Barkai [27:13] present multiple ethnographic accounts of spears or lances being used to kill elephants, particularly when the element of surprise has been achieved. Churchill [28:16] similarly found that elephants “can be dispatched by spear, provided the hunter has the time and close access necessary to repeatedly deliver well-placed stabs.” While these accounts do not specify braced lance use per se, the advantages of the braced weapon, clear from the other accounts presented here, attest to the effectiveness of pikes in bringing down large animals even when they are moving rapidly.

**Fig 6:**
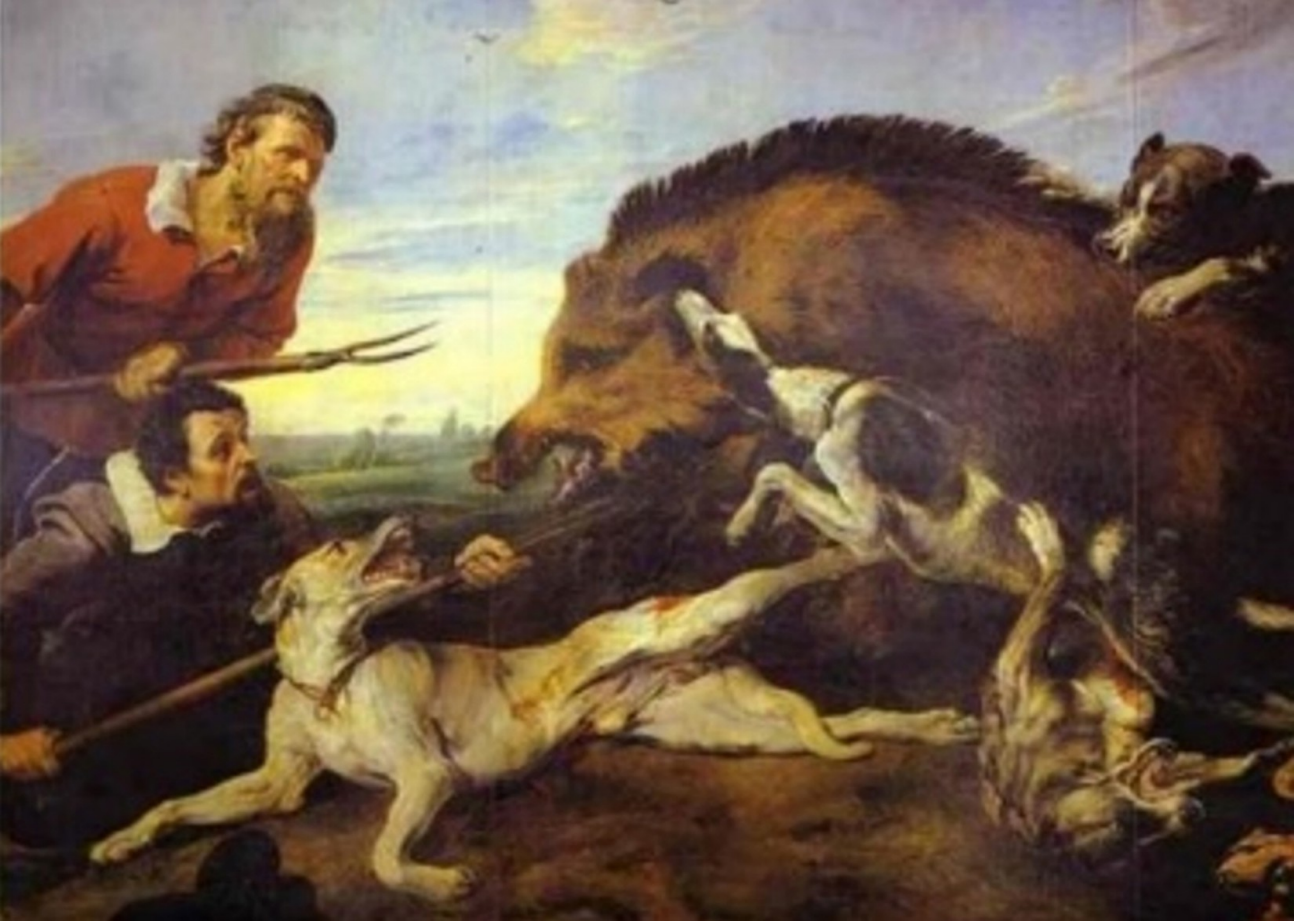
Frans Snyders, The Wild Boar Hunt, 1640. Public Domain, Palazzo Pitti, Florence, Italy.

European artwork includes numerous scenes of boar hunting with a braced weapon. Flemish artist Frans Snyders painted several studies of boar hunting including one commissioned by Philip IV of Spain for the royal hunting pavilion (Fig 6) that shows a charging boar being impaled from below by a polearm with its base likely braced in the ground. Impalement reduces the animal’s mobility, in this case allowing the thrusting spear attack from the side. The painting also illustrates the important role of dogs in megafauna hunting. The Kalinga of the Philippines were also known to use the braced spear during boar hunting in the late 19^th^ century [29:435].

Braced weapons were used in hunting unspecified bears and *Ursus arctos* identified as brown bear, Kodiak and grizzly in northern hemisphere settings. In the Yukon region of North America grizzly bear hunting with pikes is well documented. According to Ferdinand Schmitter, the Han often hunted grizzlies with this technique.

“A pike or spear is nearly always used in hunting bears. The hunter attracts the bear by making a raven-like noise, causing the bear, as the Indians say, to think the raven has discovered a dead moose. … As the bear approaches the Indian holds the spear in position, facing the bear as it draws nearer to him, and as the bear springs the Indian sticks the spear into its throat at the top of the breast-bone, at the same time shoving the handle of the pole into the ground, thus causing the bear to spear himself with his own weight. Sometimes three men hunt in this manner, two of them attacking the bear on either side as it rushes forward” [30:8].

Schmitter also noted that the Han used pikes along with dogs to hunt moose, though the technique is not described further.

The Gwich’in bear spear is three meters in length with a sharp antler tip 9 cm in length, seated in a wooden foreshaft 30 cm long which in turn rests in a wide flange that forms a socket at the distal end of a wooden mainshaft [31:8]. It was used as a pike for hunting foraging grizzlies. After thrusting the weapon into the standing bear’s chest,

“the hunter then quickly jammed the proximal end of the spear shaft to the ground and held it fast. The bear… pushed toward the man but only succeeded in further implanting the shaft deeper in the ground and in the struggle impaled itself deeper upon the spear point until it was stopped by the swelled guard portion of the distal end of the spear shaft” [31:8–9].

Frederica De Laguna [10:364] described Tlingit grizzly bear hunting on the Northwest Coast. A braced spear was used, with bows and arrows as backup weapons.

“The bear usually attacked as he emerged from the den. A party of men would wait on the slope above … armed with bows and arrows, but the bravest used spears… braced against the ground, and when the bear charged, the man would quickly jump aside, letting the bear impale himself on the spear.”

Depictions of braced weapon use in brown bear hunting in Asia are also abundant. Hallowell [9:41] notes that among the Saami of northern Europe as well as the Ainu of the western Pacific Rim, “The animal is not attacked directly, but the spear is held in reverse until the beast launches himself against the hunter and thus becomes impaled.”

Ravenstein [32:379] describes apparent pike use by Amur River peoples that involved using a cord to lift the distal portion of the weapon just as the bear approaches, timing this so the bear is impaled. There are numerous depictions of the rogatina, a pike with a metal blade that was used to hunt brown bear in Slavic countries. Through the 19^th^ century it was often used with a musket or rifle as a backup, though firearms were considered less reliable. Like the steel tipped pike, the rogatina was used for hunting and in battle [33].

**Fig 7:**
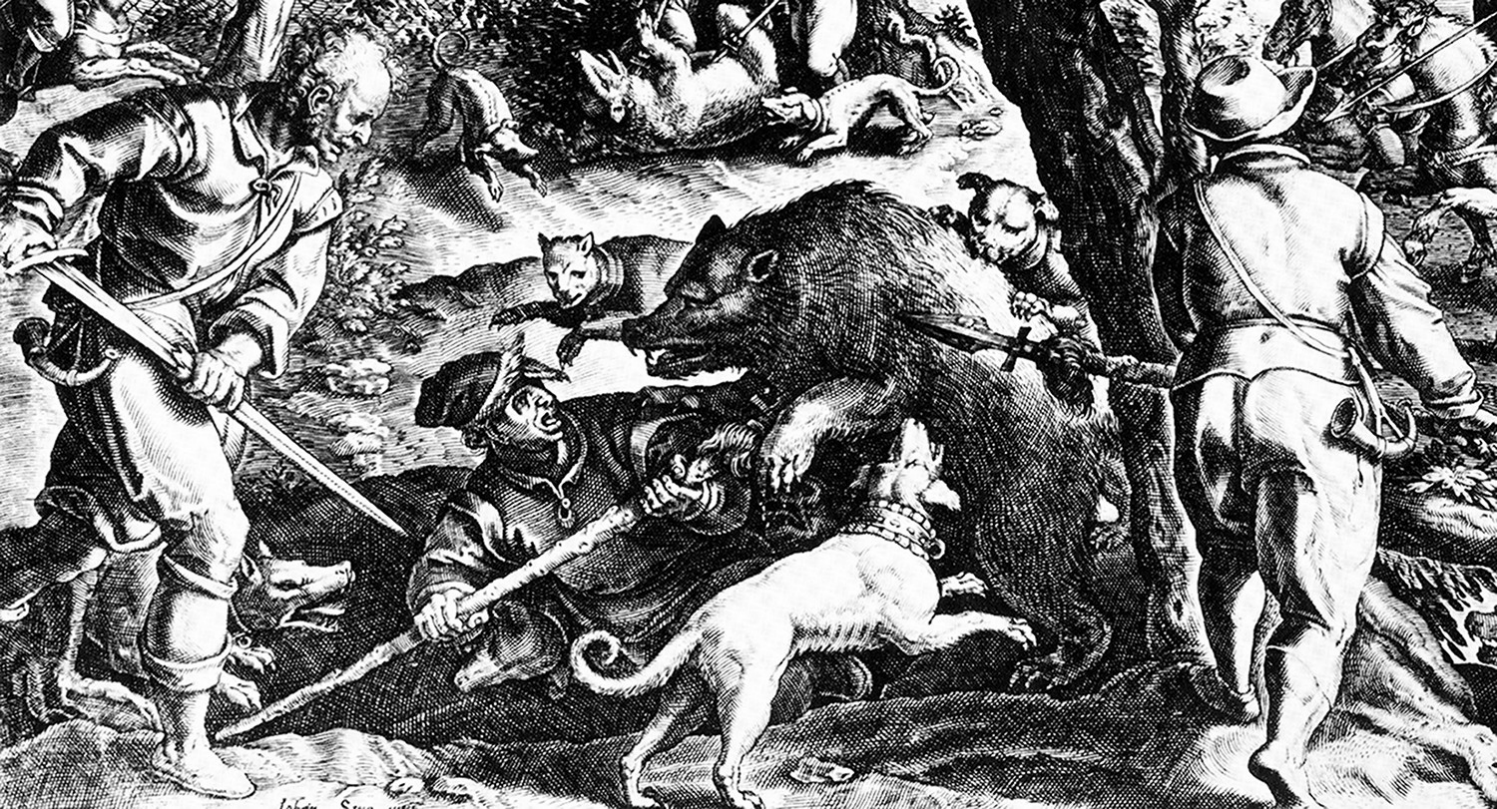
Bear Hunt engraving, 16^th^ Century Europe, Jan van der Sraet, 1578, British Museum 1957,0413.86. The bear is attacking the hunter whose pike is braced, while a second pike wielder approaches from the right.

European bear hunting with braced pikes is depicted in historical images over hundreds of years (e.g. Fig 7).

In East Africa, hunting and defense against lions sometimes involved braced weapons. Solitary Maasai lion hunters and herders used the hunting spear as a pike in guarding their communities, their family wealth (cattle), and during initiation ceremonies [34]. As described by Adamson [12:146], the Maasai

and other East African hunters have traditionally proved their manhood in this century by provoking a lion to charge and awaiting the final leap crouched upon the ground, holding a spear aslant with the butt planted to receive the shock as the lion impales himself upon the point.

Indigenous jaguar hunters in South America were known as tigreros, and in the 1800s and early 1900s they often worked for ranches hunting jaguars that preyed on livestock. Tigreros preferred impaling jaguars with stout spears over firearm use in part because they hunted in tall grass and dense foliage, and they often hunted with one or more dogs [35:259]. In 1897 journalist William Willard Howard documented Indigenous tigrero hunting on the Guajira Peninsula in Columbia and Venezuela, accompanying hunter Terife Valdez [36:387]. According to Howard, the tribes of this region hunted jaguar with a short spear, which they viewed as requiring greater skill, whereas jaguars were hunted with the more common long spear in the Amazon region of Brazil. “The hunter plants the butt of the spear-shaft in the ground, holds the point toward the jaguar at an angle of about forty-five degrees, and crouches directly behind it. The jaguar springs for the hunter, but lands squarely upon the point of the spear, while the hunter dodges to one side and rolls over out of harm’s way [36:387].” The long spear was of “hard and heavy wood” 2–2.5 meters long and 5 cm diameter with a metal head.

### 1.3 Analysis of historical data

Pikes and related braced weapons have been tipped with a wide range of materials from bone and antler to bronze and steel [17, 18, 31]. Pikes are usually longer and thicker than thrown spears, roughly 3 meters long for bear hunting but longer for military pikes used to stop horses. Spears used as a pike defensively against lions and jaguars were often under 3 meters, typically with a reinforced proximal end. Historical accounts also emphasize the pike shaft was a large investment in time and energy needing seasoned, resilient hardwood like ash or cornel [17, 19, 21]. Wooden staves suitable for pikes are often of limited availability and were traded widely.

**Fig 8:**
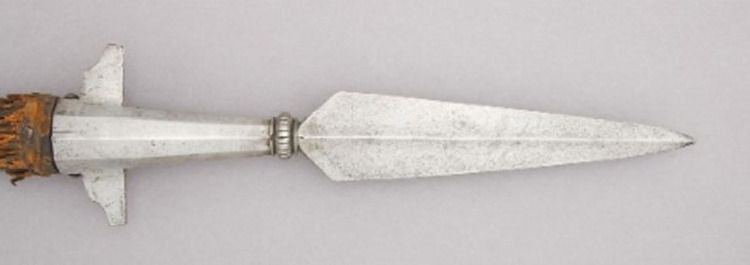
17^th^ century French boar spear, forged steel, with socket and crossguard (at left), hafted to wooden shaft. Metropolitan Museum of Art 12.141.11. Versions of this weapon are depicted in artwork shown in Figs 2 and 7, and in several entries in Table 1.

While a sharp pike tip is needed for hide entry, pikes often penetrate so deeply they put the hunter at risk of being reached by the wounded animal. Hence many pikes had a crossguard (Fig 8) or other blocking mechanism such as the wide flange on the Gwich’in bear hunting weapon, to limit wound depth [18, 23, 20:65].

**Table 1:** Historical braced weapon hunting depictions (excluding charging horse accounts). Depth-limiting mechanisms include the crossguard (CG) and flange (FLG).

Region and		Type of		>1		Brace	Second	Shaft, tip
Cultural Group	Period	Depiction	Source	Pers.	Dogs	Locat.	Weapon	Charcteristics
**Bear: Unspecified**								
Russia/European	1800s	painting	Sokolov (Fig 2)	1		stump	rifle	3 m, metal, CG
Russia/European	1843	panting	Grashov [37]	5	5	ground	axe, rifle	3 m, metal, CG
Russia/European	1800s	painting	Tikhmenev [38]	1	1	ground	rifle	3 m, metal, CG
Europe/ European	1500s	engrav.	Virgil Solus [39]	1	4	tree		<3 m, CG
Europe/European	1600s	engrav.	Van der Sraet (Fig 7)	2	4	ground	pike, swrd	~3 meters CG
Europe/Saami	1893	engrav.	Alamy img RD6EP9	1		rock	rifle	~3 meters
Europe/Saami	1920s	ethnog.	Hallowell [9]			ground		
Japan/Ainu	1920s	ethnog.	Hallowell [9]			ground		
NE Asia/Amur R.	1860s	ethnog.	Ravenstein [32]			ground		
SE Alaska/Tlingit	1800s	ethnog.	De Laguna [10]	yes		ground	arrows	barbs, tether
**Bear: Grizzly**								
Alaska/Alutiiq	1900s	Ethnog.	Washburn [11]			ground		CG
Yukon/Han	1800s	ethnog.	Schmitter [30]	yes		ground		
Alaska/Gwich’in	1860s	ethnog.	O’Brian [31]	yes		ground	arrows	3m, antler, FLG
**Boar**								
Phillipin/Kalinga	1890s	ethnog.	Moss [29]			ground		<3m metal tip
Europe/European	1640	painting	F. Snyder (Fig 6)	1	5	ground	spear	
Europe/European	1609	engrav.	Tempesta [40]	yes	yes	cliff	3 pikes	<3m, CG
Greece/Macedonia	300bc	historical	Xenophon [23]					CG
UK: Anglo-Saxon		historical	Thompson [20]					CG
**African Lion**								
E. Africa: Maasai	1900s	ethnog.	Adamson [12]	no		ground		
E. Africa: Maasai	1900s	ethnog.	Clarke [34]	no		ground		
**Jaguar**								
Brazil/Amazon	1890s	historical	Howard [36]			ground		>3m
Venezuela/Guajira	1890s	historical	Howard [36]	yes	yes	ground	no	<3m metal tip
**Carabao (Water Buffalo)**								
Phillipin/Kalinga	1890s	ethnog.	Moss, [29]			ground		<3m metal tip

These historical accounts and depictions, summarized in Table 1, detail aspects of pike use in megafauna encounters that are relevant to assessment of potential lithic pikes in archaeological contexts. Accounts of pike use against animals such as elephant and moose, lacking specific description of weapon bracing, are not included in the table.

Historical depictions of megafauna countered with braced pikes include a wide range of taxa, including warhorses (*Equus ferus caballus*), wild carabao (*Bubalis bubalis kerebau*), bear (*Ursus arctos*), boar (*Sus scrofa*), jaguar (*Panthera onca*) and lion (*Panthera leo*). These accounts indicate braced pikes were often deployed against animals much larger than humans, primarily when the animals were moving toward humans.

Triggering the animal’s charge was often key to hunting with a pike, and hunting dogs were bred and trained to drive game toward the hunter. Harassment with projectiles and other weapons was often used to provoke a charge or lunge toward the hunter, and cornering an animal was often effective for braced weapon hunting. For bear, jaguar and lion the animal either rises up or lunges at the pike wielder, whereas boars more often charge like the cavalry warhorse. The greater reach of the pike made hand thrusting more effective against taller animals defending with tusks or horns, such as the elephant. The thrust and brace technique may have been common in pike attacks on larger animals that were not moving rapidly during the initial encounter, and this technique is primarily described for bear hunting. In these accounts, animals moving quickly (horse, boar, carabao, jaguar and lion) were initially countered with the pike already in braced position.

Clovis era Pleistocene megafauna include several species that might have been hunted with pikes, such as horse, ground sloth, mammoth and bison. To the extent that people competed with megacarnivores for prey, or predators may have been driven to attack humans during periods of environmental change and resource stress, braced weaponry may have been critical for defense against *Arctodus*, *Panthera*, *Smilodon* and others, especially among highly mobile small hunter-gatherer groups. Terminal Pleistocene environmental changes that brought about extinctions [41, 42] likely increased risk for people sharing habitat with megacarnivores.

## 2. Experimental design

### 2.1 Polearms of Late Pleistocene North America

Clovis technology developed in a landscape dominated by megafauna, and it appears to have originated with highly mobile small hunter-gatherer groups [1, 43:9]. Given the historical evidence that braced weapon technology was highly effective in encounters with megafauna prey and predators, and widespread geographically spanning millennia, we now compare the hypothesized lithic pike with other polearms including thrusting spears and harpoons, examining the mechanisms involved in fluting, hafting, hide entry and impalement. With few exceptions such as fishing weir sites [44–46], sharpened wooden staves are not numerous in precontact North American contexts. Assessment of Pleistocene polearm use therefore emphasizes archaeological lithic weapon point technology and in some cases rods or points of bone, ivory or antler materials when these are preserved, though assumed wooden mainshaft characteristics are also key to design analysis, replication and experimentation [47].

For thrust or thrown spears, weapon penetration depth is limited when the animal is much larger than the weapon wielder [8, 48]. However for the braced pike, instead of the smaller human providing the force behind the weapon, impalement force is provided by the large animal’s momentum. This means deeper pike penetration unless the shaft breaks or loses its seating, or the animal stops its forward motion. Table 2 shows that there can be great variation in pike and spear hunting kinetics. These data are estimated and do not represent a comprehensive assessment of pike or spear penetration.

**Table 2:** Estimated velocity meters/second (m/sec), mass (kg) and kinetic energy (J) of various actions for braced pike impalement and projected spear penetration. Spear and dart data is approximated from Milks et al. [49] and Whitaker et al. [50]. Animal size and speed is approximated below published maximums, and mass is reduced for all but *Smilodon* due to a portion of these animals’ mass resting on the ground.

Selected Action	m/sec	kg	KE (J)
*Arctodus* charge to pike	9	400	16200
*Smilodon* lunge to pike	9	200	8100
boar charge to pike	7	120	2940
proboscidean rotating to pike	2	1000	2000
grizzly moving slowly to pike	2	300	600
atlatl propelled dart to target	34	0.3	173
spear thrust into target	4.5	65	660
spear thrown into target	16	0.8	102

In megafauna encounters with piercing shaft weapons the force of impact and penetration is the momentum provided by the moving weapon, the moving animal, or both. As Whitaker et al. [50:162] observe, kinetic energy or force of impact can be thought of as “the amount of energetic work that the projectile does as the energy is expended in cutting tissue, breaking bone and pushing tissue and bone out of the way.” A unit of kinetic energy is equal to half the mass multiplied by its velocity squared (KE = ½ m* v2). While velocity affects a projectile’s kinetic energy more than its mass does, megafauna mass values are vastly greater than the mass of a hand-held projectile. Therefore megafauna charge and lunge actions produce far greater kinetic energy than spear thrusts or projectile launches. Darts launched with an atlatl may have greater impact velocity [6, 50, 51], but transfer of kinetic energy, not momentum, is the primary determinant of wound severity [52:207]. While launched projectile spears lose force rapidly during impact, thrust spears [53:3, 54]and the braced pike sustain penetrating force after initial impact. However spear thrust penetration is expected to be less sustained than braced pike impalement if the mass of the animal is much greater than that of the weapon wielder.

When the large animal moves onto a braced stone-tipped shaft, the weapon tip transitions through stages of hide penetration, impalement, and stoppage. The lithic point may undergo changes such as breakage if it becomes embedded in bone or dense tissue during compression, as demonstrated in thrusting spear experiments [53, 54]. Points suited for tipping braced weapons may therefore have attributes that facilitate adjustment during these stage transitions.

While its length and bifacial, scalloped edge made it effective in causing massive internal injury [8, 52], the Clovis point would have also been a reliable and effective hide entry device. We envision that Clovis technology differed from harpoon points that enter the animal’s hide and then use the hide’s resilience to anchor a tether that can disable megafauna (c.f. [55:251]). In cases where the harpoon point detaches on entry, the tip’s barbs and blunt basal elements keep it anchored while the mainshaft recedes. Detachable Clovis foreshafts incorporating bone or ivory elements have also been proposed by Cotter [25] and Stanford [56] among others.

With the hypothesized lithic pike, instead of the mainshaft receding after initial penetration in the case of a harpoon, the animal continues moving onto the braced shaft, so the foreshaft components may stay bound to the mainshaft, causing enormous injury as the animal continues its forward movement. The pike shaft can impale deeply, its distal end seated in the partial socket provided by the flute. In this sense, we conceive of the Clovis point as somewhat like later pike blades of metal which often had centered basal sockets to hold the blade in place while protecting the distal wooden pike shaft end (Fig 8). However, the biface’s flute is shallow and faces outward on each face. If one face of the fluted point seats the mainshaft, the other can seat the beveled rod foreshaft. Archaeological beveled rods of bone, antler and ivory [2, 57] fit well as a splint foreshaft for Clovis points, and can be lashed to one face of the biface. The flute on the opposite face can seat the wooden pike shaft distal end. Lahren and Bonnichsen [58] and Haynes [59:122] present a different splint foreshaft design involving a wooden splint bound to the Clovis point and bone foreshaft, the latter proximally seated in a socketed spear shaft.

**Fig 9:**
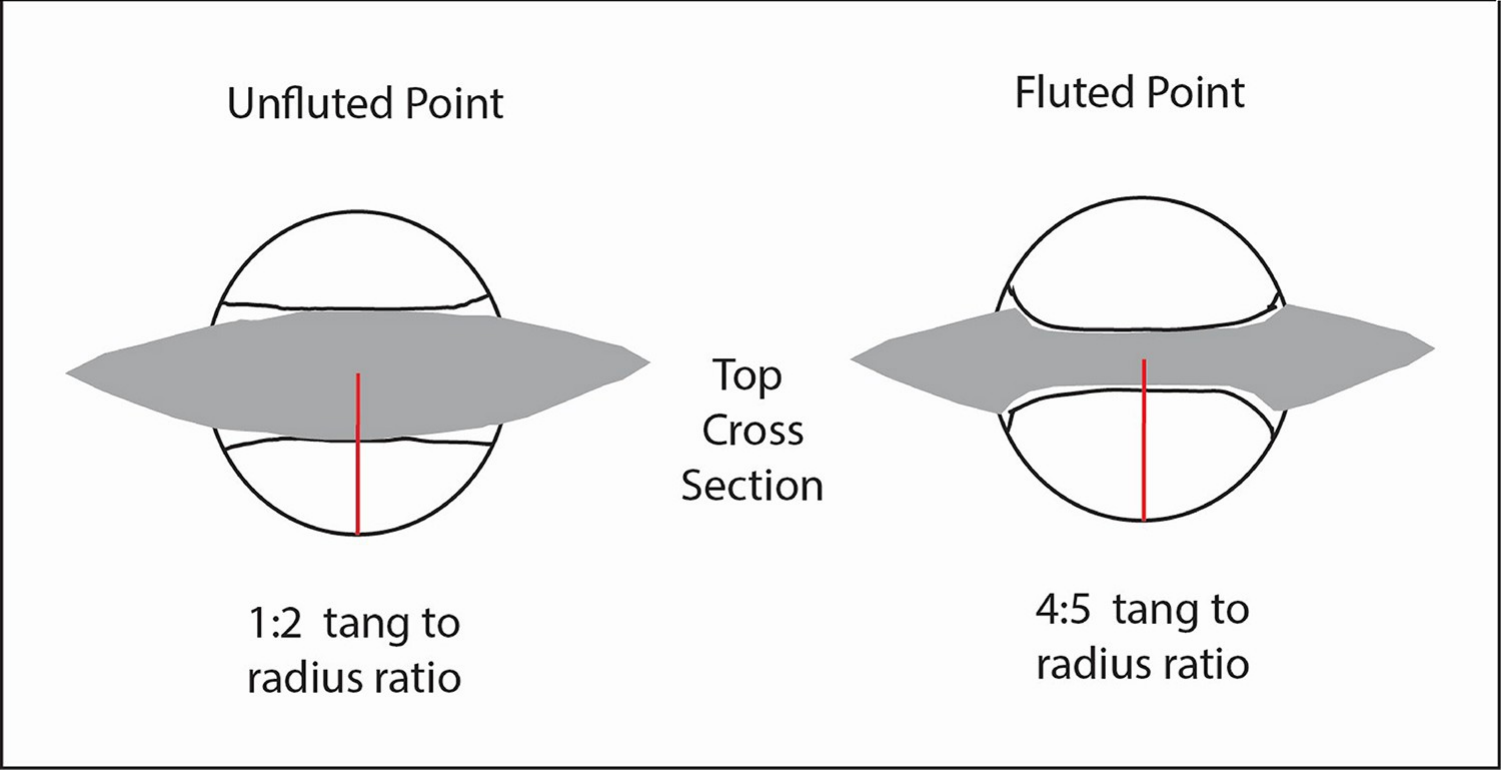
View of two bifacial points and haft elements, facing proximal direction. For a given mainshaft diameter the distal shaft end is thicker for the fluted point than for the unfluted point.

Hafted with a splint foreshaft of bone, hardwood or other resilient material, the fluted biface allows for a wider diameter distal mainshaft than hafting with an unfluted biface of similar thickness with a convex lenticular cross section because a higher proportion of shaft at the flute is wood rather than lithic material (Fig 9). For a given shaft thickness the ratio of tang thickness to shaft radius for the fluted point may be as much as 4:5, whereas the base of a similar sized unfluted biface may be closer to 1:2. A more robust wooden portion of the distal shaft end may have been preferred over a sharpened one because its sturdiness allows it to penetrate deeply enough to stop the large animal without breaking under substantial compressive force. Avoiding such attrition is especially important if the large, straight and resilient pike shaft is difficult to replace, as were many historical pikes [17, 19].

**Fig 10:**
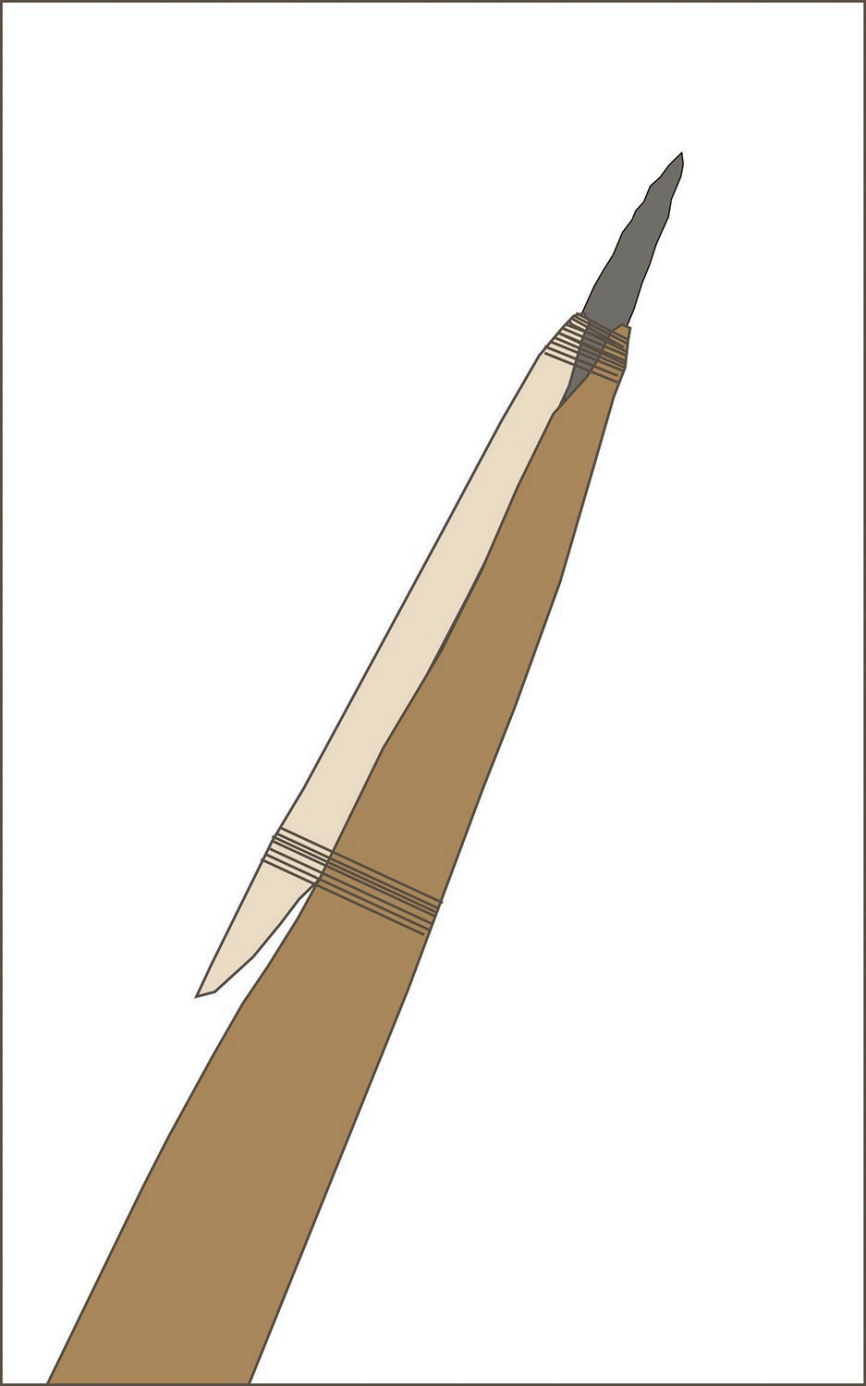
Sketch of assembled Clovis pike concept with tapered, stout mainshaft (brown) beveled bone foreshaft (beige) and lashing (black) at the fluted point (gray) and proximal rod bevel. Note the gap between the bevel face and the mainshaft while the splint foreshaft and point are hafted.

We should note here that there are no known archaeological examples of hafted Clovis points, and the wooden elements of what we assume were shaft weapons are not preserved [2, 6]. Our assumption of large shaft size is based on ethnohistoric comparisons and associations between Clovis points and megafauna. There is also much debate about the possible uses of the beveled osseous rods, sometimes found in association with Clovis points. In our hypothesized configuration the Clovis pike has three components: the wooden mainshaft tapered to a blunt tip; the sharp stone point fluted to partially seat the pike mainshaft and foreshaft; and lastly the beveled rod splint foreshaft, with lashing to hold the point and foreshaft in place (see sketch in Fig 10). It is a compact braced weapon system suitable for efficient wound entry, though it may forego some aspects of overall strength in compression as compared to those with a notch, ledge or socket for seating the point.

It is possible that the beveled rod splint haft was intended for less than maximum hafting strength. In some circumstances it may have been engineered to partially collapse under pressure. For example, as the shaft weapon moves deeper into the animal, the hafted Clovis point tip may hit bone or tissue dense enough to cause it to jam and possibly crush, burinate or suffer a bending fracture. Here the relative strength or weakness of the splint foreshaft hafting can allow for point detachment. Because the wooden mainshaft is braced and the animal’s movement is sustained, though likely decelerating, the mainshaft continues to penetrate after the jammed point recedes. Due to the massive amount of kinetic energy in megafauna momentum (Table 2), the pike tip does not need the sharpness of a Clovis point tip to penetrate deeply once the shaft has pierced the animal’s resilient hide, and a blunt mainshaft tip may increase the rate of deceleration, recalling that stoppage is one of the goals of pike use.

**Fig 11:**
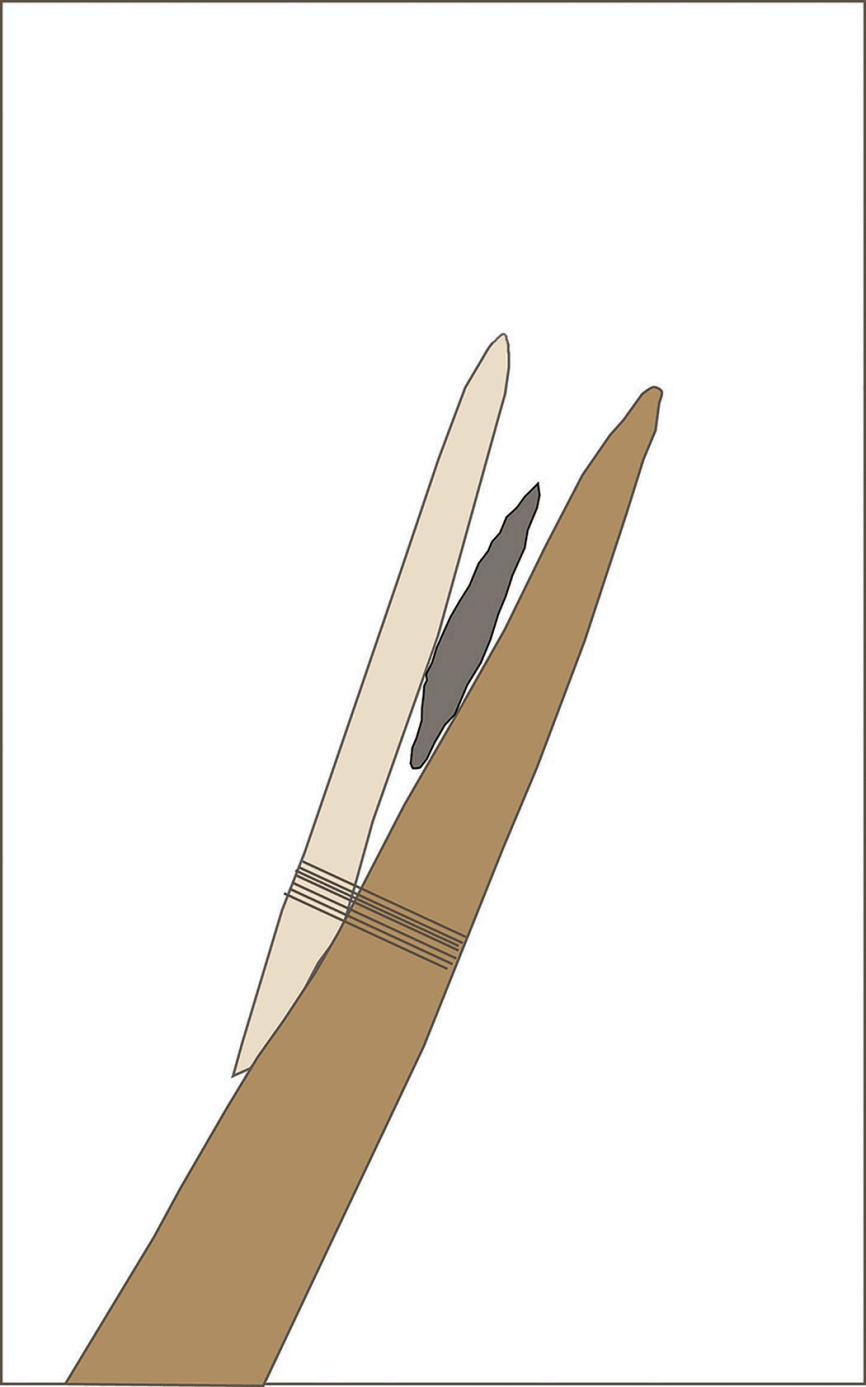
Sketch of Clovis pike with point receding after impact with bone (not shown) and engineered distal lashing rupture; rod rotation presses beveled face against the mainshaft while proximal lashing holds.

As the jammed point detaches it is likely to vector along the pike shaft in a proximal direction (Fig 11), its incurvate biface base can wedge the beveled rod apart from the mainshaft. In this manner the distal rod end hinges outward at its proximal lashing such that its proximal beveled face meets the mainshaft and resists further rotation in an outward splay. In rotation the distal rod end may serve as a second pike prong until increased pressure from the pierced animal’s forward motion causes the bone rod to detach as well (see Frison [60:156]) on the possibility of the beveled rod being both foreshaft and point). A flange could have been wrapped to the mainshaft proximal of the rod end to prevent slippage. The rotated prong tip may enter the chest cavity or other vulnerable areas even when the mainshaft has been redirected subcutaneously by the impact, increasing the chance that the weapon will cause critical injury in addition to reduction of animal kinetic energy vectored along the shaft towards the human wielder. The strain of this rotational action may be one reason beveled rods were often made of highly resilient materials such as mammoth bone and ivory. Thomas [61] raised the possibility that Clovis point design related to “shock-absorption,” or dampening of force that helps to preserve the point. Shock absorption through hafting collapse might also have increased preservation of Clovis points during high energy weapon use.

## 3.0 Materials and methods

### 3.1 Model design

We conducted three related experiments to test variables in the hypothesized splint foreshaft collapse and Clovis point recession under low velocity pike compression. We simulated this by inverting the distal pike foreshaft model in a freefall drop tower with calibrated dead weight [62, 63] using indirect numerical calculation [64]. Velocity was determined by using a known drop distance, for example 0.6m, multiplied by gravitational acceleration (9.8 m/s/s) to get 5.99m/s/s which is multiplied by 2, and the square root of this number, 3.43, is the object’s velocity at impact. The energy and momentum of the distal pike shaft was controlled by varying the released mass and the height of its initial freefall in an axial direction.

In order to assess the plausibility of pike hafting strength being set for an approximate detachment force threshold, experiment phase I determined the force needed for the point to pierce untanned cowhide (4 mm thickness). We consider this the minimum level of effectiveness for the hafted point during pike impalement. Experiment phase II tested the possibility of lashing the point and splint in place with enough strength to hold the components together during hide penetration, but to collapse when compressed against an oak plank, simulating megafauna bone, splitting the point’s lashings and allowing it to recede before compression increases enough to cause major fracturing of the biface. Experiment phase III focused on the amount of force needed to crush the Clovis point replica in low velocity impact with the oak plank, using fixed haft elements that did not collapse or allow the point to recede. This third test addresses the force against which foreshaft hafting must rupture if the fluted point is to recede and remain intact when encountering bone or other hard material during impalement.

**Fig 12:**
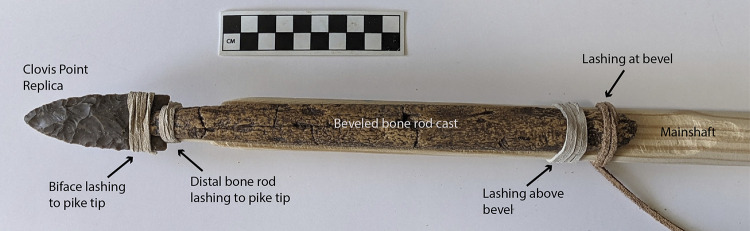
Experimental splint hafted pike with replica Clovis point tip, resin cast of East Wenatchee beveled bone rod (shown without lashing in Fig 3), yellow pine mainshaft and tanned buckskin strips for lashing.

**Fig 13:**
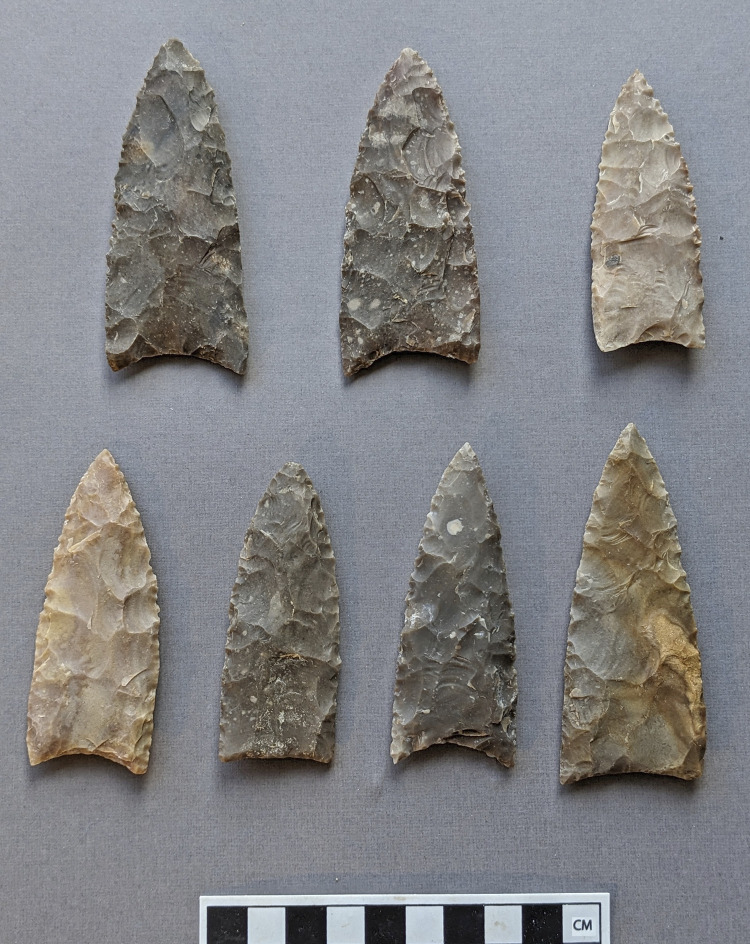
Projectile point replicas 1–3 (upper row) and 4–7 (lower row).

The characteristics of the replication model of the hypothesized Clovis pike foreshaft are shown in Fig 12. Replication involved seven Texas river cobble chert fluted points (Fig 13) made by knapper Craig Ratzat. The morphometrics of these points appear in Table 3. The points are within the metric range of Clovis points in several key attributes based on [65]. These points range from 7.8 to 9.4 cm in length and 2.9 to 3.7 cm in width, and 0.8 to 1.0 cm in thickness, with sharp tips, moderate distal blade convexity, basal fluting on both faces and basal indentation. In both experiments biface tips were sharpened with pressure flaking to where they appeared sharp but sturdy enough not to break under light pressure, and proximal lateral edges were ground to 30–35% of biface length. The two faces of each point were basally fluted to between 20% and 35% of biface length, with the average thickness at the estimated center of the flute at 68% of maximum biface thickness. Experiment trials for phase II were continued in cases where initial trials had blunted the biface tip, as the goal was to assess lashing rupture rather than point penetration.

**Table 3:** Clovis point replica measurements.

Clovis Replica No.	1	2	3	4	5	6	7
length (mm)	90.9	87.4	78.1	84	77.6	84	94.4
max. width	37	37.3	29.2	32.1	31.2	32.2	36.9
max. width height	18	28.1	14.7	18.5	25.5	25	27.5
basal width	33.8	34.9	26.9	29.4	27.7	31.3	34.7
basal concavity depth	5.5	5.1	2.8	3.7	1.7	5.5	2.3
maximum thickness	9.8	8.9	8.1	8	8.8	8.2	8
thickness mid-flute	6.5	5.7	5.2	6.5	4.8	5.3	6.3
mass (g)	33.1	25.7	19.7	24.6	23.5	22.6	29.6

For all drop tower experiments the wooden mainshaft (distal segment) was a yellow pine dowel 2.9 cm in diameter and 61 cm in length. For experiment phases I and II the distal shaft was shaped with a taper that included a planar face where the beveled splint was attached with lashing at the positions shown in Fig 12. For experiment phase III no splint was used and the fluted point was hafted in a carved notch at the distal end of the wooden shaft.

The splint foreshaft for most tests is the beveled rod epoxy resin cast shown in Fig 3, which proved to be quite durable. For replica 6 a wooden beveled splint was used, which better fit the shape of the fluting. No mastic was used for any of these experiments because of the free movement needed for the point to recede after the engineered lashing rupture, and for the splint to rotate open at a given detachment force threshold. For experiment phases I and II lashing consisted of tanned buckskin lace 2.7 mm wide and 2 mm thick (Realeather DOS50-0270 batch 2023–09) that provided strength as well as flexibility and elasticity sufficient for binding weapon components. Hafting consisted of four basal lashing wraps and five to seven proximal blade lashings as needed for biface stability. In lashing the biface to the mainshaft tip, efforts were made to minimize coverage of the distal beveled rod to facilitate rod rotation after basal lashing split during drop tower compression, simulating the force of a receding biface during pike use. For experiment phase III waxed polyester artificial sinew was used for heavier lashing, comparable to that used by Conrad et al. [47] and others.

**Fig 14:**
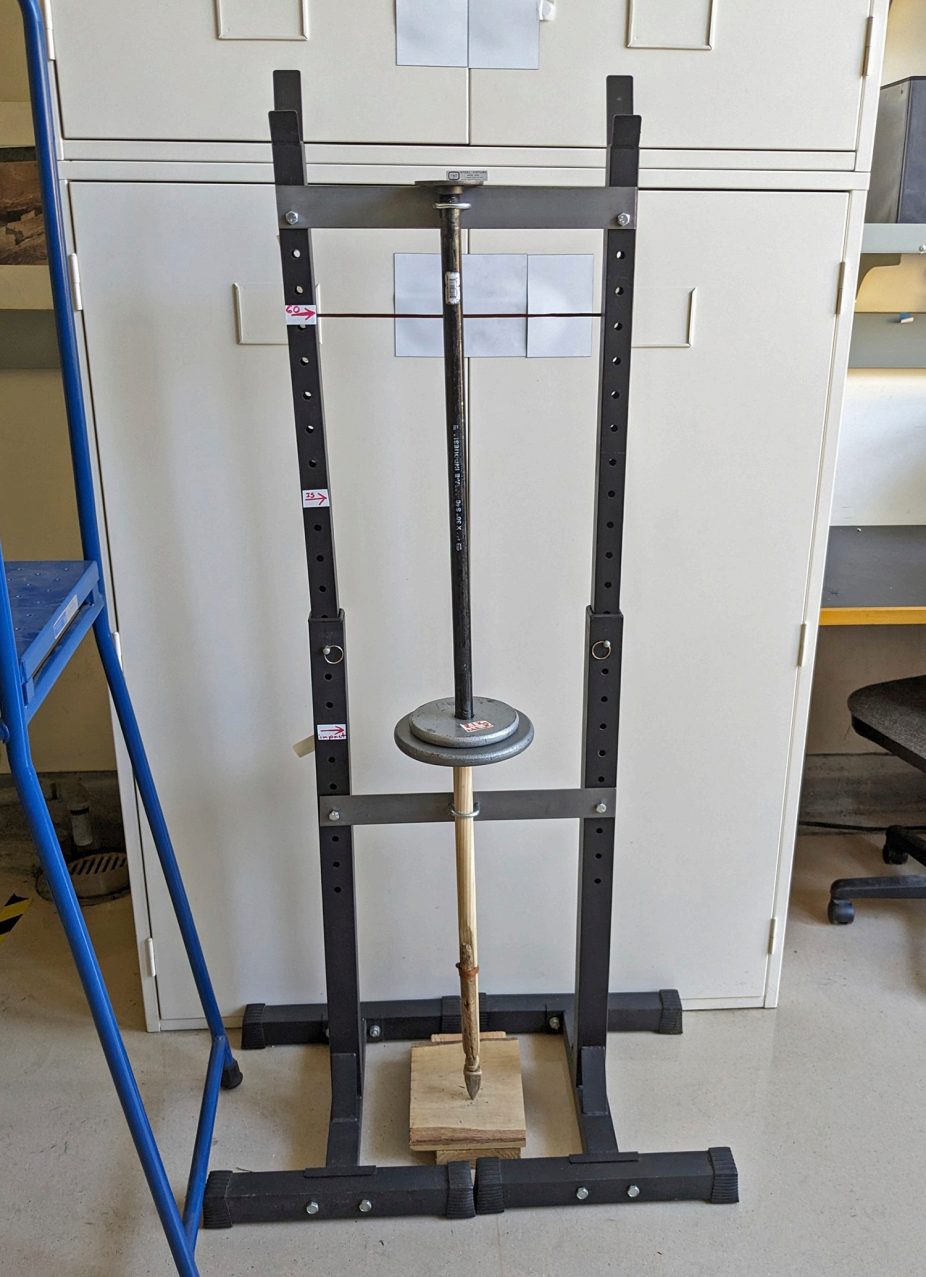
Replica 4 positioned between 8.1 kg drop weight and an oak plank in the freefall drop tower used in experiments phase I-III at Bear Bones Lab, UC Berkeley.

The freefall drop tower used in all three experiment phases consisted of a freestanding metal frame 140 cm high and 48 cm wide (not include its base) consisting of two vertical posts and two main horizontal crosspieces, at a maximum height of 180 cm (Fig 14). Additional crosspieces were added or removed as needed to provide locations for u-bolt guides or to provide greater strength for heavier dead weight use. Other freefall drop towers incorporate a guide tube (e.g. Milks [63:255]) but we chose to rely on the U-bolts attached to cross braces to minimize friction, and to allow for the width of the dead weight assembly. Weights consisted of calibrated iron plates of 2.2 and 4.4 kg positioned on a vertical threaded iron pipe 91.4 cm in length and 2.7 cm in diameter with two threaded floor flanges as end caps. The lower flange had a metal plate bolted to its face for a smooth, flat contact with the proximal wooden shaft end. A plumb bob was used to confirm the guides were positioned vertically over the proximal shaft end.

### 3.2 Experimentation

#### Phase I experiment

For the hide entry experiment, a Clovis pike splint foreshaft model was positioned at the base of the drop tower with its tip pointing downward, resting on a section of wet cow hide 22 by 38 cm supported by a block of closed cell foam 8 cm thick. Force application consisted of drop tower trials with variable mass between 1.5 kg and 5.9 kg at freefall distances ranging from 35 to 70 cm for velocities ranging from 2.66 to 3.76 meters per second. The test was repeated with increasing mass and/or drop distance at different locations on the hide until the point penetrated the hide. Tested mass and freefall distances resulted in force application of 4 joules up to 26 joules. In each case penetration was to the full width of the biface, limited by the upper flange contacting the crossbar in the axial drop tower structure.

#### Phase II experiment

The second experiment assessed lashing rupture, splint rotation at the distal bevel axis and fluted point detachment from the mainshaft and splint under low velocity compression. While the hypothesized Clovis pike would not have moved forward during megafauna impact because the shaft would be braced, the projectile point and oak plank were stationary in this experiment to simulate the effect on the haft of the animal’s forward movement. Force application consisted of a free fall drop tower test with variable mass between 3.7 kg and 12.5 kg at freefall distances 35 to 70 cm for velocities ranging from 2.66 to 3.76 meters per second. Tested mass and freefall distances resulted in force application ranging from 13 joules up to 62 joules.

#### Phase III experiment

The third experiment assessed the force necessary to crush the hafted fluted point under compression against the oak plank. This experiment used fixed hafting, consisting of a notched shaft with copious nylon sinew lashing comparable to lashing used in many other spear point experiments (e.g. [47]). Tested mass and freefall distances resulted in force application ranging from 26 joules up to 96 joules.

## 4.0 Results

The results of all trials for the three experimental phases are presented in Table 4.

**Table 4:** Results of free-fall drop tower experiments. Hyphen denotes trial with no change. KE recorded as J (Joule unit of energy) for hide penetration (HP) (phase I), lashing rupture (LR) (phase II), and point crushing (PC) (phase III) for replicas 1–7. Letters following Clovis point replica ID numbers represent separate lashing episodes for the specific biface.

	4J	6J	8J		13J	26J	39J	49J	62j	82j	96j
Phase I Hide Penetra.(HP)											
R1	--	HP									
R2	--	HP									
R2	--	HP									
R2	--	--	HP								
R4	--	--	HP								
R5a	--	--	--		--	HP					
R5b	--	--	--		--	HP					
R5d	--	--	--		HP						
R6a	--	HP									
R7a	--	HP									
Phase II Lashing Rupture (LR)											
R1a					--	--	LR				
R2					--	--	--	LR			
R4a					--	--	--	--	LR		
R4b					--	--	--	LR			
R5a					--	--	LR				
R5b					--	--	LR				
R5c					--	--	--	LR			
R6b					--	--	--	LR			
R7b					--	--	--	LR			
Phase III Point Crushing (PC)											
R2						--	--	--	--	tip	
R6a						--	--	--	--	--	PC
R4c						--	--	--	--	--	PC

### 4.1 Phase I experiment results

Of the ten hide entry trials, none of the replicas penetrated hide at under 4J. Four of the bifaces penetrated hide between 4J and 6J, including three trials with replica 2. Replica 4 pierced the hide between 6J and 8J. Each of the foregoing Clovis point replicas had a sharp, relatively fresh tip. In the case of replica 5, hide entry attempts occurred after the point had been used to test apparatus settings, and the tip had become somewhat blunt. As a result, replica 5 required between 13J and 26J to penetrate the wet hide.

### 4.2 Phase II experiment results

Nine lashing rupture trials were conducted with six replicas (1, 2, 4–7). The trials began at 13J and continued through 26J, 39J, 49J, and in one case 62J, stopping when lashing ruptured and the point receded. In some cases the point receded less than 10mm without the lashing splitting, possibly due to stretching of inadequately tightened lashing, or poor conformity of the biface base and hafting element surfaces. In two of these cases the point’s lashing was redone and the trails were resumed, repeating the freefall test at the interval that had caused the shifting. None of the 13J or 26J drop tests produced lashing rupture. Lashing ruptured on four hafted points at 39J, five at 49J, and lashing for two replicas ruptured at 62J.

**Fig 15:**
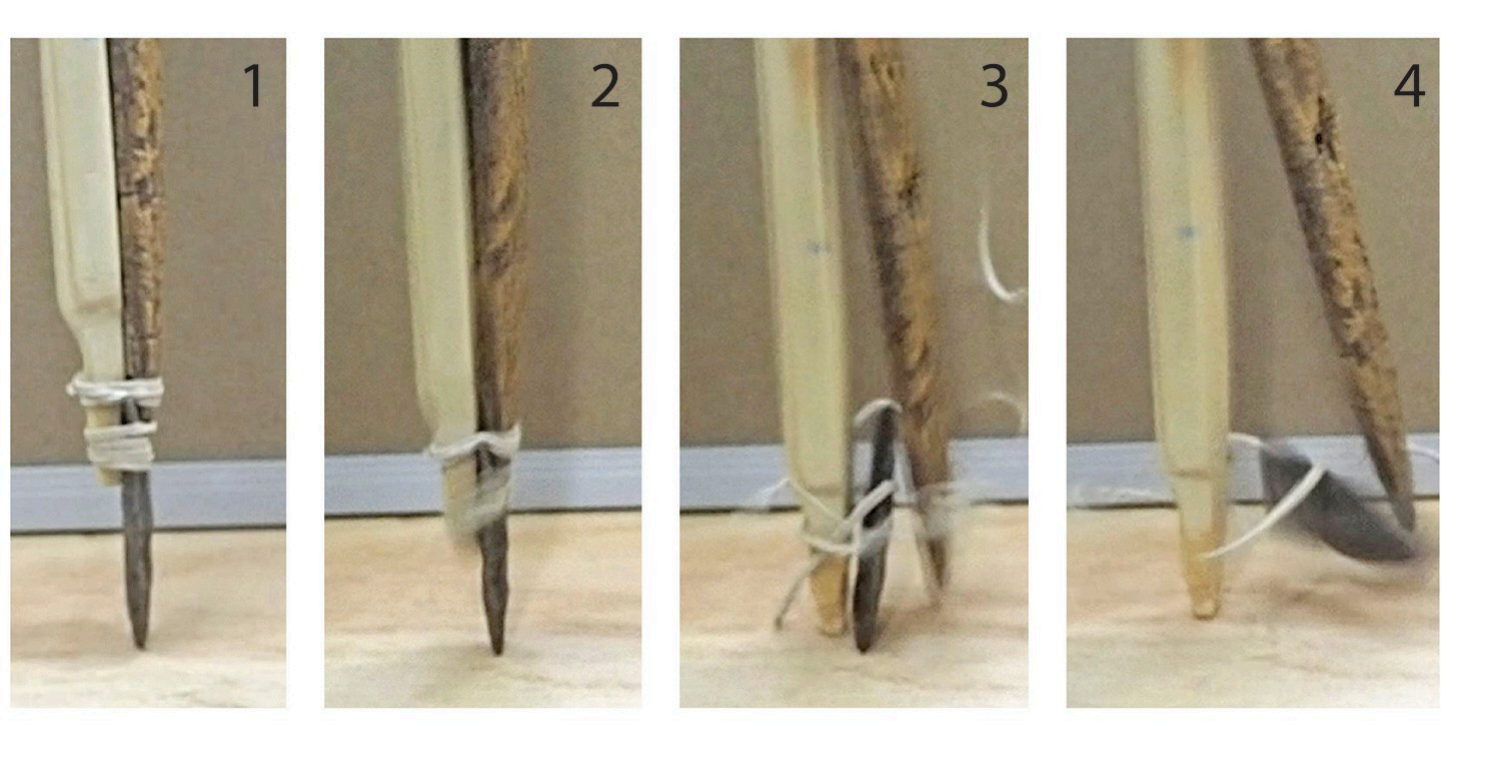
High-speed photo sequence of receding point model during ~56 J weight drop impact (11.34 kg dropped from 35 cm height) with the point tip resting on an oak plank. Image 1: before force application; Image 2: initial force presses fluted point tip into plank, point begins to recede, straining lashing; Image 3: point recedes and splits lashing, detaching as wooden shaft tip strikes plank; Image 4: distal beveled rod rotates outward, stopping when proximal bevel face (not shown) makes contact with mainshaft.

Phase II stages of lashing rupture are captured with high-speed photography (Fig 15). The force of pike compression allows the thin, fluted base of the jammed point to split or sever its lashing, wedging between the splint foreshaft and the mainshaft as it recedes. With actual impalement through the animal’s soft tissue, the shaft would not be moving, only the animal, so the fluted portion of the Clovis point would slide in a proximal direction along the distal mainshaft. At this stage the distal beveled rod may rotate away from the mainshaft distal end as it does in Fig 15 image 4, stopping when the rod’s proximal beveled face meets the mainshaft. In detaching and receding, both the point and the beveled splint remained largely intact, though minor biface tip damage was observed in some cases.

### 4.3 Phase III experiment

In examining the crushing force threshold during impact with bone (or oak plank), we found that the sturdy Clovis point undergoing slow moving impalement results in a point crushing threshold of between 62J and 82J based on three trials. However the fixed haft varied from the splint haft that is the focus of this study, and we expect the rigid haft elements would provide far less shock absorption, such that a more evenly supported biface base would withstand substantially more impact force than one that essentially rests on a flat rectangular notch base (c.f. [61]). Preliminary testing with larger mass in the splint haft configuration suggests that even when it is heavily lashed the splint haft allows the biface to recede a small amount along the mainshaft axis.

### 4.4 Summary

We found that changes in tanned buckskin lashing without mastic, such as the number of lashing wraps at a given thickness, and their positioning on the foreshaft, were sufficient to achieve a detachment force resistance threshold in the 27–62 joules range, above what is needed for hide entry with a sharpened Clovis point (between 4J and 8J), and below crushing force (above 62J). Thus, the ideal resistance force threshold needed for reliable foreshaft detachment and point recession would likely be more than 8J and less than 62J. Heavier lashing with a splint foreshaft, though not tested in this experiment, may allow for less receding that does not split the lashing but withstands higher amounts of compression without crushing.

## 5.0 Discussion and conclusions

### 5.1 Observations from experiments

These initial findings suggest that an engineered foreshaft creates conditions for collapse involving the Clovis point and splint foreshaft as a mechanism for low velocity hide entry and deep tissue damage that can adjust and continue impalement if the sharp tip meets strong resistance in harder material such as megafauna bone. When impact causes the foreshaft to collapse, this allows the less sharp but also less brittle wooden mainshaft tip to remain as the critical impalement element while the wounded animal continues movement toward the hunter. This also allows the distal beveled splint to rotate outward, which may initially serve as a second prong with an oblique trajectory corresponding to the angle of the osseous rod’s proximal bevel. Note that likely impact damage on archaeological osseous rods [2, 57] is often found at one end, suggesting use as a point or prong, and bevel fractures are also common on these artifacts, as might occur during prong rotation under compression.

We observed that the slight convexity of margins on most Clovis point forms would likely enhance proximal blade lashing rupture. The mid-lateral edge above the ground portion is often the widest part of the Clovis point. As the point receded the lashing would have been exposed to a wider blade with a sharp, bifacial edge in motion. While Werner et al. [66]- have demonstrated there is little benefit to proximal lateral edge grinding for hafting strength with fixed bifaces, close examination of the movement of the compressed splint and mainshaft relative to the biface may demonstrate a benefit from edge grinding, allowing the biface to shift under strain accumulation without immediately severing the lashing. This may have allowed dampening of impact force without full detachment in many cases.

Most critically, dynamic experiments [49, 54, 67] are needed to assess the nuances and limitations of a braced fluted point weapon system in potential megafauna encounters. Our experiment was static, yet acts of stabbing, spear thrusting and pike impalement entail many different masses moving at different velocities. These involve non-axial force and torque values that are not readily assessed with standard mechanical tests such as freefall drop towers [62:43] that entail only the axial movement of one mass. A sharp pike tip moving through the animal encounters cumulative axial resistance that is further increased by lateral or oblique force from the animal’s movement, and this is likely where much biface breakage comes from (e.g. [54:222]). Splint haft design and lashing techniques and materials will need to be further explored to account for resistance to oblique force in particular. We have not demonstrated that the foreshaft collapse can occur after hide entry in response to megafauna movement other than that directed along the axis of the weapon shaft. Examining this experimentally would likely require tests incorporating a braced pike foreshaft and a moving mass such as a block of ballistics gel.

We also recognized during experiment trials that haft lashing likely varied depending on use. For example, in defensive situations points hafted for controlled detachment below the crushing threshold could have also been readied with additional lashing that could be rapidly released. This may allow for deeper biface penetration and oblique torque resistance at the expense of point recession for biface protection and splint/prong rotation. Our preliminary experimental setup includes Clovis pikes with a wider lashing strap tightened with a half hitch that extends to a long tether along the mainshaft. Thus hafted, the foreshaft still withstands embedding in ballistics gel and requires only a light tug to loosen the added strap once the pike has entered the target, possibly pulled as the hunter moves away from the animal during initial impalement, as described for bear and jaguar encounters historically. This enhancement might also have been possible in transition from thrusting to bracing in lower velocity megafauna encounters (see Ravenstein [32:379] regarding cord use with pike in bear hunting).

### 5.2 Possible archaeological correlates of clovis pike use

Based on historical comparisons and Clovis point and osseous rod characteristics, these replicative data demonstrate the potential effectiveness of a hypothesized Clovis pike incorporating the fluted point, beveled osseous rod, and a robust pike mainshaft, though only two of the three weapon components are represented at Late Pleistocene sites. We rely on the foregoing ethnohistorical accounts for our assessment that pikes would have best suited Clovis hunters in megafauna encounters.

While detachment means the biface is more likely to remain intact, detached points may be lost if the pierced animal is not killed in the encounter or after tracking. Thus Clovis points as receding pike tips may help account for the high archaeological frequency of Clovis point isolates [68, 69:270]. These individual points found away from sites could represent locations where animals expired sometime after being wounded elsewhere by lithic pikes. Similarly, braced weapon use might account for several sites, including Naco, Lehner, Miami, and Escapule [48:2, 69:287, 70], where megafauna appear to have died with embedded complete points, apparently away from hunters who otherwise might have butchered them and recovered the detached points.

Pike use also fits with associations between Clovis points and osseous rods, though these rods are known from earlier non-Clovis contexts in North America, Asia and Europe as well [71]. Blackwater Draw Locality 1 may hold the best archaeological example of Clovis point and splint foreshaft use, represented in the mammoth block of Cotter’s 1936 excavation [25]. Likely weapon elements were found with a small number of apparent butchering tools among the bones of a single Columbian mammoth. Saunders and Daeschler [72] found that butchering was done on some of the carcass after it had stiffened. However the presence of two complete Clovis points and two complete bone rods seems a high rate of valuable tool loss for low intensity scavenging. It is more likely that the Clovis points and beveled rods were left in wounds made during an unsuccessful hunt that occurred elsewhere, with the mammoth eventually expiring at the draw. These artifacts may represent two splint-hafted Clovis pikes whose foreshafts collapsed and points receded during impalement. The weapon elements likely went undiscovered by the hunters who later found the stiffened carcass at the water source and butchered portions of it, including limbs and some rib areas. Haynes [59] notes that the depositional settings of natural death sites of modern African elephants are similar to the settings of mammoth sites in North America, and Blackwater Draw is no exception. Taphonomic processes may have dispersed one of the bone splint and biface pairs, while the close proximity of the other fluted point and beveled rod suggests these are closer to their original position within the partially butchered carcass.

Fracture patterns in Clovis points may also correspond to the proposed Clovis pike configuration. Huckell [54:222] notes a high frequency of breakage of thrusting spear points. The splint foreshaft/receding fluted point design may have addressed this problem. Damage reduction from slight receding (frame 2 in Fig 14) may have been intended, with the flute and lashing keeping the point in alignment for continued penetration after minor impact cushioning.

According to Kilby et al. [53:9] there is a lower frequency of impact-fractured points at Clovis kill sites than at post-Clovis kill sites. Eren et al. [48] argue that the low frequency of impact fractures on Clovis points indicates they were not frequently used for hunting megafauna such as proboscideans, and instead were used as butchering tools. We suggest the low frequency of impact fractures may partly result from Clovis point use in a collapsing foreshaft configuration. Further archaeological indication of reduced damage during Clovis point and splint foreshaft use may also appear in the findings of Buchanan et al. [73], who found that resharpening was not a significant source of Clovis-point variation in points sourced to three prominent Midwestern quarries.

Researchers often note the high degree of uniformity in Clovis point bases despite great variability in biface size. As David Meltzer [69:305] writes, this implies “that the hafts were more difficult or time-consuming to make, or the haft material was harder to come by.” The reason may be that the weapon tip had to have a balance of tip sharpness needed for hide entry, blade width and scalloping for maximum wound effect, tapering for deep impalement, and robusticity needed to maintain shaft integrity throughout impalement compression. Reliability and durability through ongoing hunting of charging megafauna with a lithic pike may have benefited from biface base characteristics enabling the receding mechanism in the splint foreshaft. The high quality, seasoned wood necessary for pike resilience means the shaft may have been a bigger investment in time and energy than the Clovis point itself, so maintaining and preserving it was critical. Use of high quality lithic material [74] for finely crafted lanceolate fluted points would have also been a worthwhile investment, as the consequences of tip deflection at the hide entry stage are dire when the hunter is relying on the pike to stop charging megafauna.

Although damage clearly from Clovis points is exceedingly rare on skeletal material [59], we might expect such damage to be present where pike impact is more likely, such as the anterior ribs for bear, lion, sabertooth and sloth, as these megafauna are more likely to have charged or lunged forward. Modern elephants are known to sweep their tusks from side to side in defense [22], which might allow two or more hunters to approach and set pikes sequentially from opposite sides. Pike response to mammoth tusk sweep could explain the positioning of points in the Naco mammoth as well [70, 75]. We might not expect to find archaeological beveled rods immediately adjacent to Clovis points because of separation during detachment and continued animal movement, but general proximity as seen at Blackwater Draw would be expected in carcasses of animals that escaped hunters after an attack.

Given that Native people were present in North America millennia before the appearance of fluted points, why didn’t the Clovis innovation occur at an earlier time? The majority of accounts of historical pike use involve charging megafauna, where the weapon is shown to be highly effective. While many of these encounters were triggered by humans, circumstances were very different during the Late Pleistocene, when megafauna were predominant. Small group mobility may have been limited in this setting, particularly when resource stress brought about shifts in prey choices and competition over kills among *Arctodus*, *Smilodon* and *Panthera*. The Clovis innovation may have involved enhancement of existing unfluted braced weaponry as a reliable, ready response to these changes, providing greater safety and hunting success for highly mobile small groups.

### 5.3 Projectiles, thrust spears and pikes

The lithic pike weapon system would have had distinct advantages over thrust spears and projectiles in many encounters with Pleistocene megafauna that were generally faster moving and much larger than humans. Pikes are shown to be especially effective at protecting the hunter while critically injuring the confronted animal. The finely crafted biface and foreshaft with the sturdy mainshaft can together convert the momentum of the moving bison or mammoth into impalement (resistance force) that may be an order of magnitude greater than the penetrating force of a spear thrust or launch. If the pike is securely braced, its force resistance is the same whether it is wielded by a juvenile defending against *Arctodus* or an adult hunting a proboscidean, though deploying the lithic pike in many encounters would have necessitated enormous skill and resolve acquired through experience. And while the pike’s major strength is in bracing to receive a charge, when used as an extended thrusting spear its longer reach makes the pike useful for spearing large animals while keeping a few steps further from moving tusks or horns. Braced weapons can also be used to limit megafauna mobility to allow for well-placed thrusts with additional pikes or spears, providing the time and safety needed for the post-disadvantaging coup de grace that thrusting weapons are well suited for [28].

According to Churchill, hunting with a hand-delivered thrust or thrown spear is dependent on terrain for containing prey (i.e. disadvantaging), while the atlatl-thrown dart requires less dependence on terrain due to its greater range. He notes “[T]he effective exploitation of a wide range of terrestrial mammals characteristic of modern humans occurred after the advent of efficient projectile weapons [28:11].” Similarly Binford [76] saw prey selection as dependent on the ability of humans to overtake game with speed, launched weapons, corralling, or the element of surprise (Binford 1984). These findings have been influential in Clovis studies, where disadvantaging containment in wetlands or other terrain settings is often seen as necessary to reduce megafauna prey mobility for spear use. However the ethnohistorical information we have presented on braced weapon use demonstrates that the behavior of several megafauna taxa can be influenced toward aggressiveness sufficient for the animal to charge or lunge at the hunter, setting the stage for effective pike use as we have proposed with our Clovis pike model. The pike is similarly effective against megafauna predators that may be aggressive toward humans because they see humans as prey or as their competitors. Disadvantaging megafauna prey or predators by triggering their charge eliminates the spear’s constraint of dependence on terrain, with important implications for the evolution of social hunting.

Lastly, being larger and heavier than most thrown or thrust spears, pikes take up a larger portion of a portable toolkit, and pike shaft harvest and seasoning may be more time consuming than for smaller weapons. This may have been made up for if pike shaft use manifested the multifunctionality of Clovis weaponry that researchers often emphasize [48, 77]. Pike shafts are often long enough and stout enough to serve as small tipi poles, with implications for hide shelter [43:9] and fire maintenance among highly mobile indigenous groups in open country. This and other pole uses (travois, tanning racks etc.) would mean carried pike shafts could be useful during the wood seasoning period [17:324] necessary for shaft resilience prior to pike deployment, particularly in grasslands of central and western North America, where wood for structures may not be readily available at short term camps. Adzes identified at several Clovis sites [3, 78:139] would have been suitable for shaping these multipurpose staves.

### 5.4 Conclusions

The Clovis pike may have been an innovation in weapon technology especially suited to highly mobile small hunter-gatherer groups encountering numerous megafauna species during a period of massive environmental change in Late Pleistocene North America. Fluted point use did not outlast megafaunal extinctions by many generations. Yet ethnohistorical records demonstrate that braced piercing weapons were in use thousands of years later in North America and on other continents, and unfluted pikes could have been used prior to the Late Pleistocene. As archaeologists move forward with research on braced weapons we may find that Clovis technology provides valuable insight into other braced weapon strategies that were perhaps somewhat common during the Paleolithic, given millennia of coexistence between humans and now extinct megafauna. The possibility of lithic points and osseous rods as pike tip elements offers new perspective on the dimensions of social hunting and human interaction with megafauna in the Pleistocene.

## References

[1] Waters MR. Late Pleistocene settlement and exploration of the Americas by modern humans. Science. 2019: 365. doi: 10.1126/science.aat5447 31296740

[2] Bradley, BA, Collins, MB, Hemmings, A. *Clovis Technology*. International Monographs in Prehistory, Archaeological Series 2010;17. Ann Arbor, Michigan.

[3] Jennings TA, Smallwood AM. The Clovis record. SAA Archaeo. Record. 2019;19(3), 45–50.

[4] Story BA, Eren MI, Thomas K, Buchanan B, Meltzer D. Why are Clovis fluted points more resilient than non-fluted lanceolate points? A quantitative assessment of breakage patterns between experimental models. Archaeometery; 2019;61(1);1–13. 10.1111/arcm.12407

[5] Milks A, (2020) A Review of ethnographic use of wooden spears and implications for Pleistocene hominin hunting. Open Quaternary 2020;6(12) 1–20. 10.5334/oq.85

[6] Coppe J, Lepers C, Rots V (2022) Projectiles under a new angle: a ballistic analysis provides an important building block to grasp Paleolithic weapon technology. Journal of Archaeological Method and Theory 2022;29: 1131–1157. 10.1007/s10816-022-09551-z

[7] Eren MI, Story B, Perrone A, Bebber M, Hamilton M, Walker R, et al. North American Clovis point form and performance: An experimental assessment of penetration depth. Lithic Technology. 2020; 263–282. 10.1080/01977261.2020.1794358

[8] Wilkins J, Schoville BJ, Brown KS (2014) An Experimental Investigation of the Functional Hypothesis and Evolutionary Advantage of Stone-Tipped Spears. *PLOS ONE*. 2014;9(8): e104514. 10.1371/journal.pone.0104514 25162397 PMC4146534

[9] Hallowell AI. Bear ceremonialism in the northern hemisphere. American Anthropologist. 1926 (28); 1–175.

[10] De Laguna F. Under Mount Saint Elias: The history and culture of the Yakutat Tlingit. Washington, D.C.; Smithsonian Institution Press; 1972.

[11] Washburn SL. Social life of early man. London; Routledge; 1961. 10.4324/9781315017761

[12] Adamson HE. Anthropology, The study of man. 4^th^ edition McGraw-Hill;1972. https://archive.org/details/anthropologystud00hoebrich

[13] Fedje D, Mackie Q, McLaren D, Christensen T. A projectile point sequence for Haida Gwaii. In Carlson R, Magne M, editors. Projectile point sequences in Northwestern North America. Burnaby; Simon Fraser University Press; 2008; 19–40.

[14] McLaren DR, Wigen, Mackie Q, Fedje D. Bear hunting at the Pleistocene/Holocene transition on the northern Northwest Coast of North America. *Canadian Zooarchaeology* 2005; 22: 3–29.

[15] Byram S, Lightfoot KG, Sunseri J. Projectiles or pikes? Clovis point attributes and braced weapon use. Soc. American Archaeo. 2023. https://core.tdar.org/document/474372/projectiles-or-pikes-clovis-point-attributes-and-braced-weapon-use

[16] Baldino J, McKinny S, Taylor J, Wilson M, Buchanan B, Walker RS, et al. North American Clovis point form and performance V: An experimental assessment of spear thrusting penetration depth and entry wound size. Lithic Technology 2023; 1–16. doi: 10.1080/01977261.2023.2270255

[17] Markle MM. The Macedonian sarissa, spear, and related armor.” American Journal of Archaeology. 1977 81; 323–39. 10.2307/503007.

[18] Matthew C. *An invincible beast*: *Understanding the Hellenistic pike-phalanx at war*. Barnsley, South Yorkshire: Pen and Sword Military 2015.

[19] Sekunda Nikolas (2001): The Sarissa. Folia Archaeologica 2001;23: 13–41.

[20] Thompson L. *Ancient weapons in Britain*. Barnsley Yorkshire; Pen and Sword Military; 2005.

[21] Spaulding TMA, Manuscript of Sir John Smythe’s Certain discourses concerning formes and effects of weapons. 1590. *The Papers of the Biblio*. *Soc*. *of America*, 1937; 31(2):180–184. Univ. Chicago Press, http://www.jstor.org/stable/24296540.

[22] Scullard EC. The Elephant in the Greek and Roman World. Cornell Univ. Press. 1974. ISBN 13: 9780801409318 https://archive.org/details/elephantingreekr0000scul

[23] Marchant EC, Bowersock GW. Xenophon, On hunting. Constitution of the Athenians. 1925. https://www.perseus.tufts.edu/hopper/text?doc=Xen.+Hunt.+5.13

[24] Lyman RL, O’Brien MJ, Hayes V. A Mechanical and Functional Study of Bone Rods from the 24-Roberts Clovis Cache, Washington, U.S.A. Journal of Archaeological Science. 1998;25: 887–906. 10.1006/jasc.1997.0270

[25] Cotter JL. The occurrence of flints and extinct animals in pluvial deposits near Clovis, New Mexico, part IV: Report on the excavations at the gravel pit in 1936. Proc. of the Philadelphia Acad Nat. Sci. 1937 (89); 1–16. https://www.jstor.org/stable/4064241

[26] Trautmann TR. *Elephants and kings*: *An environmental history*. Univ. Chicago Press; 2015. 10.7208/chicago/9780226264530.002.0003

[27] Agam A, Barkai R. Elephant and mammoth hunting during the Paleolithic: A review of the relevant archaeological, ethnographic and ethno-historical records. *Quaternary*. 2018; 1:1–28 10.3390/quat1010003

[28] Churchill SE. Weapon technology, prey size selection, and hunting methods in modern hunter-gatherers: Implications for hunting in the Palaeolithic and Mesolithic. In Peterkin GL, Bricker HM, Mellars P, editors, Hunting and animal exploitation in the later Palaeolithic and Mesolithic of Europe. 1993;4: 11–24. 10.1525/ap3a.1993.4.1.11

[29] Moss TE. The Calinga. American journal of clinical medicine. 1904–07;14(4); 427–435.

[30] Schmitter F. Upper-Yukon native customs and folk-lore. Smithsonian Miscellaneous Collections. 1910; 56(4).

[31] O’Brien T. *Gwich’in Athabaskan implements*: *History*, *manufacture and usage according to Reverend David Salmon*. Fairbanks; Univ. Alaska Press 2011. https://www.jstor.org/stable/jj.1176787

[32] Ravenstein EG. *The Russians on the Amur*. London; Trubner Press, 1861.

[33] Kirpichnikov AN. The Ancient Russian Weapons. 1971.

[34] Clarke J. *Save me from the lion’s mouth*: *Exposing human-wildlife conflict in Africa*. Struik: Random House; 2013. ISBN 13: 9781920544751

[35] Fernández JA. Doñana: Spain’s wildlife wilderness. New York; Taplinger Publishing Company 1975: 259.

[36] Howard WW. Hunting the jaguar in Venezuela” The Century; 1897 (54): 387.

[37] Grashov O. The Tsarevich Alexander Nikolaevich on a bear hunt on the outskirts of Moscow. 1843. Hulton Fine Art Collection, Getty Images, editorial # 599935163.

[38] Tichmenev EA. Bear hunt. 1934. Hulton Fine Art Col., Getty Img. editorial # 520725707.

[39] Solis V. The bear hunt. (engraving) Metmuseum.org 700629. 16^th^ Century.

[40] Tempesta, A (1609) A boar hunt, plate 3, Hunting Scenes VI, Achenbach Foundation, San Francisco, Acc # 1963.30.36455.

[41] Beschta RL, Ripple WJ. Large predators and trophic cascades in terrestrial ecosystems of the western United States. *Biological Conservation*. 2009;142 (11): 2401–2414. 10.1016/j.biocon.2009.06.015

[42] DeSantis LRG, Crites JM, Feranec RS, Fox-Dobbs K, Farrell AB, Harris JM et al. Causes and Consequences of Pleistocene Megafaunal Extinctions as Revealed from Rancho La Brea Mammals. Current Biology 2019(29); 2488–2495. doi: 10.1016/j.cub.2019.06.059 31386836

[43] Amick D (2017) Evolving views on the Pleistocene colonization of North America. *Quaternary International*. 2017;431:125–151. 10.1016/j.quaint.2015.12.030.

[44] Byram RS. Brush fences and basket traps: The archaeology and ethnohistory of tidewater weir fishing on the Oregon coast. 2002 Ph.D. dissertation, Univ. Oregon. https://works.bepress.com/byram/5/ Accessed 20 May 2024

[45] Erlandson JM, Tveskov MA, Byram RS. The development of maritime adaptations on the southern Northwest Coast of North America. Arctic Anthropology, 1998 35(1); 6–22. http://www.jstor.org/stable/40316453. Accessed 20 May 2024

[46] Moss M. L. (2011). *Northwest Coast*: *Archaeology as deep history*. Univ, Press Colorado 2011. http://www.jstor.org/stable/jj.3850487

[47] Conrad G, Hough S, Baldino J, Gala N, Buchanan B, Walker R, et al. Clovis bone versus stone weapon tip penetration: thinking about relative costs and benefits, experimental assumptions, and archaeological unknowns at Sheriden Cave, Ohio, U.S.A. Journal of Archaeological Science: Reports, 2023;52, 104295. 10.1016/j.jasrep.2023.104295

[48] Eren MI, Meltzer DJ, Story B, Buchanan B, Yeager D, Bebber MR (2021) On the efficacy of Clovis fluted points for hunting proboscideans. Journal of Archaeological Science. Reports; 2021;39(103166):1–14.

[49] Milks A, Champion S, Cowper E, Pope M, Carr D. Early spears as thrusting weapons: Isolating force and impact velocities in human performance trials, Journal of Archaeological Science: Reports 2016; 191–203. 10.1016/j.jasrep.2016.09.005

[50] Whittaker JC, Pettigrew DB, Grohsmeyer RJ (2017): Atlatl dart velocity: Accurate measurements and implications for Paleoindian and Archaic archaeology. PaleoAmerica. 2017;3: 161–181. doi: 10.1080/20555563.2017.1301133

[51] Callahan E. A mammoth undertaking. Bul. Primitive Tech. 1(7): 23–39.

[52] Salem PE, Churchill SE. Penetration, tissue damage, and lethality of wood- versus lithic-tipped Projectiles. In Radu I, Sano K, editors, *Multidisciplinary Approaches to the Study of Stone Age Weaponry*. Series on Vertebr. Paleobio. and Paleoanth. 2016. doi: 10.1007/978-94-017-7602-8_14

[53] Kilby JD, Surovell TL, Huckell BB, Ringstaff CW, Hamilton MJ, Haynes CV. Evidence supports the efficacy of Clovis points for hunting proboscideans. J. Archaeo. Sci.: Reports, 2022; 45:103600. 10.1016/j.jasrep.2022.103600.

[54] Huckell BB. The Denver elephant project: A report on experimentation with thrusting spears. Plains Anthropol 1982;27: 217–224. 10.1080/2052546.1982.11909079

[55] Dixon JE. *Bones*, *Boats & Bison*: *Archeology and the First Colonization of Western North America*. Albuquerque: University of New Mexico Press; 1999. 9780826321381

[56] Stanford DJ. Foreshaft sockets as possible clovis hafting devices. Current Research in the Pleistocene. 1996;13: 44–46.

[57] Sutton MQ. Paleoindian-Era Osseous Rods: Distribution, Dating, and Function, PaleoAmerica, 2018;4; 183–201. 10.1080/20555563.2018.1525600

[58] Lahren L, Bonnichsen R. Bone Foreshafts from a Clovis Burial in Southwest. Montana Science. 1974;186: 147–150. doi: 10.1126/science.186.4159.147 17744223

[59] Haynes G. The Early Settlement of North America. Cambridge; University Press. 2002. ISBN 0 521 81900 8

[60] Frison GC. The Sheaman Site: A Clovis Component. In: Frison GC, Stanford DJ, editors. The Agate Basin Site: A Record of Paleoindian Occupation of the Northeastern High Plains. New York: Academic Press; 1982. pp. 143–157. doi: 10.2307/j.ctv2sx9g56

[61] Thomas K, Story BA, Eren MI, Buchanan B, Andrews BN, O’Brien MJ, et al. (2017) Explaining the origins of fluting in North American Pleistocene weaponry. J. Archaeo. Sci. 2017;81: 23–30. 10.1016/j.jas.2017.03.004

[62] Chadwick EK, Nicol AC, Lane JV, & Gray TG. Biomechanics of knife stab attacks. *Forensic Science International*. 1999;105(1), pp.35–44. doi: 10.1016/s0379-0738(99)00117-6 10605074

[63] Milks A. Lethal threshold: The evolutionary implications of middle Pleistocene wooden spears. Ph.D. Diss, Univ. College London Inst. Archaeo.; 2018.

[64] Li H., Chen W, H. Factors influencing impact force profile and measurement accuracy in drop weight impact tests. Int. J. of Impact Engineering. 2020; 145:103688. 10.1016/j.ijimpeng.2020.103688.

[65] Morrow JE, Morrow TA. Geographic variation in fluted projectile points: a hemispheric perspective. American Antiquity. 1999:215–30. 10.2307/2694275

[66] Werner A, Krame A, Reedy C, Bebber MR, Pargeter J, Eren MI. Experimental assessment of proximal-lateral edge grinding on haft damage using replicated late Pleistocene (Clovis) stone projectile points.” Archaeo. and Anthro. Sciences. 2019;11: 5833–5849. 10.1007/s12520-017-0594-2

[67] Gala N, Mika A, Wilson M, Williams J, Buchanan B, Walker RS, et al. Experimental assessment of obsidian versus chert lanceolate projectile point durability and robusticity: Semi-static fracture strength and dynamic impact. Archaeometry. 2022;64:1307–1324. 10.1111/arcm.12787

[68] Anderson DG, Miller DS, Yerka SJ, Gillam JC, Johanson EN, Anderson DT, Goodyear AC, Smallwood AM. PIDBA (Paleoindian Database of the Americas) 2010: Current Status and Findings. Archaeo. of Eastern N. America 2010; 38: 63–90.

[69] Meltzer DJ (2021) First peoples in a new world: Populating ice age America. New York; Cambridge 2021. doi: 10.1017/9781108632867

[70] Ballenger, Jesse A M. Reassessing Naco, Arizona’s first Clovis site. *Archaeology Southwest*. 2009;23(3) 7–9. https://www.archaeologysouthwest.org/pdf/arch-sw-v23-no3.pdf

[71] Wygal BT, Krasinski KE, Holmes CE, Crass BA, Smith KM (2022) Mammoth ivory rods in eastern Beringia: Earliest in North America. American Antiquity. 2022;87(1):59–79. doi: 10.1017/aaq.2021.63

[72] Saunders JJ, and Daeschler EB. “Descriptive analyses and taphonomical observations of culturally-modified mammoths excavated at ‘the gravel pit,’ near Clovis, New Mexico in 1936.” Proc. Acad. Natural Sci. of Philadelphia. 1994 145: 1–28. https://www.jstor.org/stable/4064981

[73] Buchanan B, Eren MI, Boulanger MT, O’Brien MJ. Size, shape, scars, and spatial patterning: A quantitative assessment of late Pleistocene (Clovis) point resharpening. J Archaeol Sci Rep. 2015;3:11–21. doi: 10.1016/j.jasrep.2015.05.011

[74] Buchanan B, Hamilton MJ, Kilby JD, Gingerich JA. (2016) Lithic networks reveal early regionalization in late Pleistocene North America. Journal of Archaeological Science. 2016;65, 114–121. 10.1016/j.jas.2015.11.003

[75] Haury EW, Antevs E, Lance JF. Artifacts with Mammoth Remains, Naco, Arizona. American Antiquity. 1953;19: 1–14. 10.2307/276409

[76] Binford LJ Faunal remains from Klasies River mouth. New York: Academic Press; 1984.

[77] Smallwood AM, Jennings TA. Experiments as analogues: use-wear analysis of Clovis bifaces from the Gault Site, Texas. In Kornfeld M, Huckell BB editors, *Stones*, *bones*, *and profiles*. Boulder: Univ. Colorado Press. 2016:103–126. http://www.jstor.org/stable/j.ctt1ch764r

[78] Eren MI, Desjardine A. Flaked stone tools of Pleistocene colonizers: Overshot flaking at the Red Wing Site, Ontario. In Jennings T, Smallwood A, editors. *Clovis*: *current perspectives on technology*, *chronology and adaptations*. College Station; Texas A&M Univ. Press; 2014: 109–120. muse.jhu.edu/book/38207.

